# Feasibility of Reducing and Breaking Up University Students' Sedentary Behaviour: Pilot Trial and Process Evaluation

**DOI:** 10.3389/fpsyg.2021.661994

**Published:** 2021-06-10

**Authors:** Oscar Castro, Ineke Vergeer, Jason Bennie, Stuart J. H. Biddle

**Affiliations:** ^1^Physically Active Lifestyles Research Group (USQ-PALs), Centre for Health Research, University of Southern Queensland, Springfield Central, QLD, Australia; ^2^Centre for Behaviour Change, University College London, London, United Kingdom

**Keywords:** college students, sitting time, COM-B model, theoretical domains framework, implementation research

## Abstract

**Background:** Accumulating high levels of sedentary behaviour has been linked to poor health outcomes. This study examined the feasibility and preliminary, short-term effects of a theory-based intervention aimed at reducing total and prolonged sedentary behaviour in University students.

**Design:** A quasi-experimental (pre-post) pilot study. Methods: Nine ambulatory undergraduate students (Mean age = 22 ± 2.32) participated in a one-on-one session, including an educational component around the health effects of sedentary behaviour and three distinct activities (feedback, “pros and cons” exercise, and suggested behaviour change strategies). In addition, automated daily text messages targeting sedentary behaviour were sent for 6 days (four messages per day at fixed intervals). The Behaviour Change Wheel framework guided the intervention design process. Outcomes were assessed over 6 days in pre- and post-intervention periods and included accelerometer-based (activPAL) and self-reported (Nightly-Week-U) total sedentary time, as well as accelerometer-based number of steps and prolonged sedentary time. Students completed a process evaluation interview upon completing the trial.

**Results:** From pre- to post-intervention, there was a significant reduction in accelerometer-based total and prolonged sedentary time during weekend days. In addition, there was a significant increase in accelerometer-based standing time and stepping during weekend days. There were no statistically significant changes in accelerometer-based sedentary time, standing time or number of steps during weekdays. Process evaluation results indicated that the intervention and its assessment is feasible. Reductions in sedentary time were likely to be mediated by positive changes in the student's reflective and automatic motivation.

**Conclusions:** Findings from this small, short-term intervention suggest that a single one-on-one session, together with automated text messages, may help University students reduce sedentary behaviour and enhance movement during weekend days. Additional strategies to maximise the intervention effects are discussed (e.g., establishing a collaboration with University staff, introducing sit-to-stand desks, and/or facilitating social support). A randomised control trial assessing sedentary behaviour over a longer period is needed to adequately study the intervention's effectiveness.

## Introduction

Sedentary behaviour refers to any waking activity involving low energy expenditure and where sitting, reclining, or lying is the dominant posture (Tremblay et al., 2017). Accumulating high levels of sedentary behaviour has been linked to negative physical and mental health outcomes (Biswas et al., 2015; Zhai et al., 2015; Patterson et al., 2018). As a result, in addition to promoting aerobic and muscle-strengthening activities, public health guidelines now recommend individuals minimise the amount of time spent sedentary, as well as break up long periods of sedentary behaviour (Department of Health, 2014; Department of Health, Physical Activity, Health Improvement and Protection, 2019).

The majority of sedentary behaviour and public health research to date have focused on desk-based office workers (Gardner et al., 2016), as most of their working hours are spent sitting. Similar to office workers, University students also spend most of their waking hours behind a desk, either studying or attending lectures (Castro et al., 2020a). Thus, more recently, there has been an increased number of intervention studies specifically targeting University student's sedentary behaviour. Cotten and Prapavessis (2016) conducted a randomised control trial and found small-to-moderate effects favouring the effectiveness of a text message-based intervention in increasing University students' non-sedentary behaviours (especially light-intensity physical activity). In a pilot randomised control trial, Sui and Prapavessis (2018) provided evidence for the potential of an intervention to increase break frequency during occupational (student) sedentary behaviour. Some other interventions (i.e., Jerome et al., 2017; Mnich et al., 2019) have focused on introducing environmental changes. Jerome et al. (2017) tested the effects of introducing sit-to-stand desks into a University classroom on student's sitting and standing behaviours. Their findings support sit-to-stand desks as an approach to reducing sedentary behaviour in University classrooms. Mnich et al. (2019) found that placing decisional cues in open study areas (i.e., posters and table plaques with phrases such as “Standing up means studying easier!”; “Be smart, stand up!”) is an effective strategy to nudge students to use existing sit-to-stand desks, decreasing University student's sedentary behaviour and promoting active alternatives.

While the above-mentioned studies were somewhat successful in changing their respective target behaviours in the short-term, a limitation is that interventions were often not informed by a particular theory of behaviour change or, at least, theoretical guidance was not explicitly reported. To develop effective behaviour change interventions, however, it is important to have a theoretical understanding of what behaviour is and how behaviour change works, so the relevant mechanisms of change can be appropriately targeted (Michie et al., 2008). The Behaviour Change Wheel (BCW) is a theory-driven framework that provides a systematic way of developing interventions (Michie et al., 2014). The Capability, Opportunity, Motivation, Behaviour (COM-B) model is at the heart of the BCW and posits that changing behaviour involves changing one or more of the following: capability, opportunity, and motivation (i.e., is greater capability, more opportunity, and/or stronger motivation required to achieve change?). Within these three components, the model specifies further subdivisions: capability is divided into physical capability (physical skills) and psychological capability (the capacity to engage in the necessary thought processes, such as knowledge or reasoning); opportunity is divided into physical opportunity (afforded by the environment) and social opportunity (afforded by the cultural milieu that dictates the way we think about things); and motivation is divided into reflective motivation (involving conscious plans and evaluations) and automatic motivation (involving emotional responses as well as impulses/habits resulting from associative learning). As an optional step within the BCW, the Theoretical Domains Framework (TDF) can be used to provide a more detailed understanding of the COM-B components (Cane et al., 2012). The TDF is an integrative framework of behaviour change constructs that was developed to provide a theory-based, comprehensive approach to identify influences of behaviour. An overview of the 14 TDF domains linking to the COM-B components is available as online Supplementary Material (Table 1).

**Table 1:** The Theoretical Domains Framework (v2) with definitions and component constructs Michie et al., 2014.

**COM-B components**	**TDF domains linking to COM-B components**	**Definition**	**Constructs**
Psychological capability	Knowledge	An awareness of the existence of something	Knowledge (including knowledge of condition/scientific rationale) Procedural knowledge Knowledge of task environment
Psychological capability	Memory attention and decision processes	The ability to retain information, focus selectively on aspects of the environment and choose between two or more alternatives	Memory Attention Attention control Decision making Cognitive overload/tiredness
Psychological capability	Behavioural regulation	Anything aimed at managing or changing objectively observed or measured actions	Self-monitoring Breaking habit Action planning
Physiological capability	Skills	An ability or proficiency acquired through practise	Skills Competence/ability/skill assessment Practise/skills development Interpersonal skills Coping strategies
Reflective motivation	Intentions	A conscious decision to perform a behaviour or a resolve to act in a certain way	Stability of intentions Stages of change model Trans theoretical model and stages of change
Reflective motivation	Goals	Mental representations of outcomes or end states that an individual wants to achieve	Goals (distal/proximal) Goal priority Goal/target setting Goals (autonomous/controlled) Action planning Implementation intention
Reflective motivation	Beliefs about consequences	Acceptance of the truth, reality, or validity about outcomes of a behaviour in a given situation	Beliefs Outcome expectancies Characteristics of outcome expectancies Anticipated regret Consequents
Reflective motivation	Optimism	The confidence that things will happen for the best or that desired goals will be attained	Optimism Pessimism Unrealistic optimism Identity
Reflective motivation	Beliefs about capabilities	Acceptance of the truth, reality or validity about an ability, talent or facility that a person can put to constructive use	Self-confidence Perceived competence Self-efficacy Perceived behavioural control Beliefs Self-esteem Empowerment Professional confidence
Reflective motivation	Social/professional role and identity	A coherent set of behaviours and displayed personal qualities of an individual in a social or work setting	Professional identity Professional role Social identity Identity Professional boundaries Professional confidence Group identity Leadership Organisational commitment
Physical opportunity	Environmental context and resources	Any circumstance of a person's situation or environment that discourages or encourages the development of skills and abilities, independence, social competence and adaptive behaviour	Environmental stressors Resources/material resources Organisational culture/climate Salient events/critical incidents Person × environment interaction Barriers and facilitators
Social opportunity	Social influences	Those interpersonal processes that can cause individuals to change their thoughts, feelings, or behaviours	Social pressure Social norms Group conformity Social comparisons Group norms Social support Power Intergroup conflict Alienation Group identity Modelling
Automatic motivation	Emotion	A complex reaction pattern, involving experiential, behavioural, and physiological elements, by which the individual attempts to deal with a personally significant matter or event	Fear Anxiety Affect Stress Depression Positive/negative affect Burn-out
Automatic motivation	Reinforcement	Increasing the probability of a response by arranging a dependent relationship, or contingency, between the response and a given stimulus	Rewards (proximal/distal, valued/not valued, probable/improbable) Incentives Punishment Consequents Reinforcement Contingencies Sanctions

A critical step within the BCW framework involves using the COM-B model (and possibly the TDF) to identify what needs to change for the behaviour to shift in the desired direction. Drawing a parallel with the medical field, the BCW describes this step as “behavioural diagnosis.” It is hypothesised that a good behavioural diagnosis is more likely to lead to effective interventions, because it is clear which mediators of change need to be targeted. While this might seem like an obvious step, interventions are not always designed based on a thorough analysis of the behaviour and its determinants, but on personal experience (“common sense”) or a favoured theoretical approach (West and O'Neal, 2004). Based on the behavioural diagnosis results, the BCW provides guidance regarding which intervention functions and associated Behaviour Change Techniques (BCTs) are likely to bring about change for a given behaviour. These BCW-indicated strategies are drawn from an analysis of the literature and an expert consensus exercise, which were part of the BCW development process and resulted in an indicative mapping of intervention types and COM-B/TDF targets (Michie et al., 2011, 2014). For example, if physical and social opportunity are thought to be important for the performance of a given behaviour (behavioural diagnosis), the BCW provides a broad indication as to the likely effectiveness of interventions based on “environmental restructuring,” “coercion,” and/or “enablement.” Each of these intervention types might be enacted by a range of related BCTs (e.g., environmental restructuring might be enacted by using the BCTs “adding objects to the environment,” “social support,” and/or “restructuring the physical/social environment”).

Once the intervention has been designed, a key step before the start of a large-scale trial is pilot testing (Steckler et al., 2002). Conducting a pilot trial is helpful for several reasons, such as identifying recruitment or budget problems, optimising the intervention content and mode of delivery, informing on the accuracy of the measurement tools, and/or estimating the intervention's effect size (Thabane et al., 2010). At this stage, a process evaluation can have an important role in understanding the feasibility of the intervention and refining its design and evaluation (Oakley et al., 2006). The updated UK Medical Research Council (MRC) guidelines provide directions on how to structure the process evaluation of pilot trials (Moore et al., 2015). This framework recommends: (i) assessing the acceptability of implementation structures; (ii) testing intermediate mechanisms (to get a better understanding of the pathways between intervention and outcomes); and (iii) identifying contextual factors associated with variations in outcomes. All these are critical to inform the intervention's future scale-up efforts.

The aims of the present study were 2-fold: (i) to explore the preliminary, short-term effects of a BCW-informed intervention to reduce total and prolonged sedentary behaviour among a small sample of University students (outcome evaluation); and (ii) to assess the feasibility of the intervention and its intended assessment (process evaluation).

## Materials and Methods

### Study Design

Data for this quasi-experimental (one group pre-test/post-test) trial were collected in February 2020. Participants underwent a 6 day baseline assessment of sedentary time, took part in a one-on-one intervention session, and completed a 6 day post-intervention assessment immediately following the one-on-one session. In addition, a process evaluation interview was conducted at the end of the 6 day post-intervention assessment. Both pre- and post-intervention assessments included 4 weekdays and 2 weekend days. Participants provided informed written consent and were offered a $30 gift voucher upon completion of the trial. Ethical approval was obtained from the University of Southern Queensland's (USQ) Human Research Ethics Committee (No. H19REA314).

### Participants

Participants were eligible if they were aged 18 years or over, ambulatory, and studied on-campus and full-time at the USQ's Faculty of Business, Education, Law and Arts. Participants were invited to participate via an announcement on the University-wide online portal. Interested participants contacted OC through email to arrange the initial on-campus meeting.

### Intervention

The first stage within the BCW consists of identifying, in terms of the relevant COM-B and TDF components, what needs to change in the person and/or the environment to achieve the desired behaviour. This process, known as “behavioural diagnosis,” is typically informed by an analysis of local sources and/or the scientific literature (Michie et al., 2014). For the present study, two previous qualitative studies with University students were used to draw an accurate picture of sedentary behaviour and its influences specifically for this population subgroup, highlighting relevant areas for change (Deliens et al., 2015; Castro et al., 2020b). It is worth noting that one of the two qualitative studies (Castro et al., 2020b) that informed the behavioural diagnosis was conducted with students at the same University as the present study (i.e., different samples from the same population).

The behavioural diagnosis results are detailed in Table 2 (first column: “Behavioural diagnosis using TDF domains linking to COM-B components—What needs to change?”). A list of BCW-indicated BCTs likely to be effective in changing sedentary behaviour was generated based on the behavioural diagnosis and discussed among the study authors. For example, a common finding in previous qualitative studies is the idea of a goal conflict between carrying out the University tasks and reducing and breaking up sedentary behaviour (which relates to “reflective motivation” within the COM-B and, more specifically, “belief about consequences” within the TDF). Thus, it was surmised that something that needs to change is the students' perception that reducing and breaking up sedentary behaviour during academic activities will disturb their work and concentration. Following with the above example, the BCW suggests a series of BCTs that are thought to be potentially useful when changing “beliefs about consequences,” such as “information about health consequences,” “credible source,” or “framing/reframing.” Therefore, these techniques were incorporated into the intervention content, as part of both the one-on-one session and the text messages. A similar process was followed with the rest of the behavioural diagnosis results (Table 2).

**Table 2:** Behavioural diagnosis for target behaviour “reducing and breaking up sedentary time”, along with intervention functions, behaviour change techniques, intervention strategies, and mechanisms of action.

**Behavioural diagnosis using TDF domains linking to COM-B components—What needs to change?**	**Intervention functions^a^**	**Behaviour Change techniques (BCT v1)^b^**	**Intervention strategies employed (BCT implementation)**	**Potential mechanisms of action^c^**
**Psychological capability**				
Knowledge - Know that accumulating high levels of sedentary behaviour has negative physical and mental consequences, and that prolonged sedentary time is particularly detrimental to health - Know when and for how long break up sedentary time, including which activities constitute an effective break from sitting	Education, training	Information about health consequences, information about social and environmental consequences, instruction on how to perform a behaviour	- Raising awareness about the risks of sedentary behaviour through infographics and copies of public health guidelines (text messages and one-on-one session—introduction) - Provide instructions on break frequency and duration, including strategies to break up sedentary behaviour (text messages and one-on-one session—introduction and 3rd activity: suggested strategies)	Knowledge, attitude towards the behaviour, belief about consequences, intentions
Memory, attention and decision processes - Notice and remember to reduce and break up sedentary behaviour	Enablement, Environmental restructuring	Self-monitoring of behaviour, adding objects to the environment, prompts/cues	- Prompt the participant to identify and reduce “mindless sedentary behaviour.” This is, daily activities that could be easily done standing or walking, but that are undertaken in a sitting position as this is the default position (e.g., waiting in the bus stop) (text messages and one-on-one session −3rd activity: suggested strategies) - Prompt the participants to set an alarm for every 30 min or employ other similar strategies to break up sedentary behaviour (e.g., use playlists with a set duration, use of activity trackers) (text messages and one-on-one session −3rd activity: suggested strategies) - Send daily reminders to break up and reduce sedentary behaviour via automated text messages (text messages) - Provide visual cues (posters) reminding participants to reduce and to break up their sedentary behaviour (one-on-one session −3rd activity: suggested strategies)	Behavioural regulation, behavioural cueing
Behavioural regulation - Set specific goals in relation to reducing and breaking up sedentary behaviour - Establish a method to monitor sedentary behaviour	Education, training, enablement	Self-monitoring of behaviour, feedback on behaviour, goal setting (behaviour), action planning	- Provide the participant with individually tailored feedback on sedentary time in order to guide goal-setting (one-on-one session −1st activity: normative feedback) - Encourage self-monitoring of behaviour by using a smartphone, a tracking device or a workbook with daily checklists (text messages and one-on-one session −3rd activity: suggested strategies) - Encourage goal setting and action planning to specify when, where, and how participants will reduce and break up sedentary behaviour (implementation intentions) (text messages and one-on-one session −3rd activity: suggested strategies and optional goal-setting) - Provide generic tips to reduce and break up sedentary behaviour and invite participants to identify strategies specific to their circumstances (text messages and one-on-one session −3rd activity: suggested strategies)	Goals, behavioural regulation, motivation, feedback processes, skills
**Reflective motivation**				
Beliefs about consequences - Reinforce the physical and mental health benefits of reducing and breaking up sedentary behaviour - Challenge the perception that reducing and breaking up sedentary behaviour during private academic activities will disturb the student's work and concentrationIntentions - Develop intentions to reduce and break up sedentary behaviour during private academic activities	Education, persuasion	Information about health consequences, information about social and environmental consequences, credible source, framing/reframing, instruction on how to perform the behaviour, social comparison	- Present data supporting the idea that reducing and breaking up sedentary behaviour has a positive impact on health, as well as on cognitive processes related to academic performance (e.g., attention levels, mental fatigue) (text messages and one-on-one session—introduction) - Suggest that the participant might think of taking short breaks as a way to “refresh” his attention and improve performance (rather than procrastination) (text messages and one-on-one session—introduction) - Provide guidance on how to work efficiently while reducing and breaking up sedentary behaviour (e.g., highlight tasks that can be undertaken standing up, or recommend strategies to assist the students in getting back to their work quickly after the break) (one-on-one session—introduction and 3rd activity: suggested strategies) - Raise awareness about the fact that University students typically show higher levels of sedentary behaviour compared to the general population and thus should pay special attention to their sedentary behaviour patterns (text messages and one-on-one session—introduction)	Knowledge, attitude towards the behaviour, belief about consequences, intentions, skills, social/professional role and identity
**Automatic motivation**				
Reinforcement - Establish routines and habits to break up sedentary behaviour	Environmental restructuring, training, incentivisation	Habit formation, behavioural practise/rehearsal, self-monitoring of behaviour, self-reward	- Prompt rehearsal and repetition of the target behaviour in the same context repeatedly so that the context elicits the behaviour (e.g., suggest the participant to consistently break up sitting while studying in his room, or stand up while having coffee every morning) (text messages and one-on-one session −3rd activity: suggested strategies) - Send daily reminders to break up and reduce sedentary behaviour via automated text messages (text messages) - Prompt self-reward if there has been progress in reducing and breaking up sedentary behaviour (one-on-one session −3rd activity: suggested strategies)	Behavioural cueing, reinforcement
**Social opportunity**				
Social influences - Promote social acceptability for reducing and breaking up sedentary behaviour	Environmental restructuring, restriction	Social support (unspecified), information about others' approval, identification of self as role model	- Inform the participant that other students approve and encourage reducing sitting and taking breaks (text messages and one-on-one session—introduction) - Encourage the participant to involve other students when reducing and breaking up sedentary behaviour and “spread the message” (text messages and one-on-one session −3rd activity: suggested strategies) - Suggest that the participant's own behaviour may be an example for other students to reduce and break up their sedentary behaviour (one-on-one session −3rd activity: suggested strategies)	Subjective norms, social influences, self-image

a *The Behaviour Change Wheel describes nine potential intervention functions. This is, broad categories of means by which an intervention can change behaviour, including education, training, persuasion, incentivization, coercion, restriction, modelling, environmental restructuring, and enablement (Michie et al., 2014)*.

b *A Behaviour Change Technique (BCT) is an “active ingredient” of change and is defined as an “observable, replicable, and irreducible component of an intervention designed to alter or redirect causal processes that regulate behaviour” (Michie et al., 2014). The Behaviour Change Technique Taxonomy version 1 (BCTTv1) includes 93 BCTs grouped within 16 categories and can provide a greater level of intervention detail for synthesis, comparison, and replication of studies*.

c *The Theory and Technique Tool specifies 26 different mechanisms of action, defined as processes through which behaviour change occurs (Michie et al., 2018)*.

Once the intervention content was developed, team decisions were made regarding the intervention delivery, taking into account practical criteria (e.g., time and resources available) as well as previous intervention studies targeting sedentary behaviour. For example, Cotten and Prapavessis (2016) found that sending regular text-messages is an effective strategy to reduce University students' sedentary behaviour. In addition, previous studies provide support for the efficacy of a single face-to-face intervention session in reducing sedentary behaviour in older adults (Gardiner et al., 2011; Fitzsimons et al., 2013). Thus, we decided to use a one-on-one session, together with automated text-messages, as the mode of delivery for the intervention.

The one-on-one session lasted ~45 to 60 min and was facilitated by OC, who is a Ph.D. candidate with a background in psychology and has completed different qualitative and quantitative research courses as part of his bachelor and master's degree studies. The session was structured around an intervention workbook, which included an introduction with key concepts/health effects of sedentary behaviour and three distinct activities: (i) review of accelerometer-assessed sedentary behaviour from the 6 day baseline assessment (including normative feedback); (ii) “pros and cons” activity to help students reflect on the idea of changing their sedentary behaviour; and (iii) suggested strategies to reduce and break up sedentary time (Supplementary File 1—intervention workbook). The suggested strategies were developed using the behavioural diagnosis results (Table 2), as well as “general tips” for changing sedentary behaviour found in previous intervention studies (Neuhaus et al., 2014; Maylor et al., 2018). Students were also introduced to several freely available resources that might facilitate reducing and breaking up sedentary time (e.g., posters and other visual cues, mobile and computer apps).

In addition to the individual session, participants received a series of daily text-messages during the 6 day post-intervention assessment. A total of 24 messages (four per day) were sent daily at fixed intervals: morning (10:00 am), afternoon (14:00 pm and 17:00 pm) and evening (20:00 pm). These served two purposes: (i) to act as prompts/reminders for the students to reduce and break their sedentary time; and (ii) to reinforce the key messages delivered during the face-to-face session (Supplementary File 2—list of intervention messages). The text messages were sent automatically via an SMS scheduling app and covered four broad areas: nudge messages (i.e., generic break up prompts such as “If you've been sitting for more than an hour consider getting up and move! Try walking around or doing some light stretching”), health-related messages (e.g., “Walking burns 5 times the calories that sitting does. Take every opportunity to walk around!”), psychological well-being and productivity messages (e.g., “Breaking up sitting time with short walking breaks has been shown to counteract mental fatigue, in comparison with continuous sitting”), and suggested strategies to reduce and break up sitting (e.g., “You can use habit formation strategies to change your sitting patters. For example, try to consistently pair standing breaks with daily habits such as texting on the phone or drinking coffee”). The specific wording and “tone” of the messages were developed following evidence-based recommendations for effective sedentary behaviour messaging: Alley et al. (2019) suggest messages may be more effective at reducing sedentary behaviour if they are achievable, specific and recommend healthy alternatives to sitting (e.g., standing or being active). In addition, previous intervention studies using sedentary behaviour messaging with University students were used to help generate ideas on the SMS content (Cotten and Prapavessis, 2016; Mnich et al., 2019).

With regards to the style of delivery for the BCTs forming the intervention, some of them were delivered in a passive (educational) way (e.g., “information on health consequences”). This applied to the first introductory section of the intervention session, as well as the text messages, and was based on the behavioural diagnosis results (which highlighted that sedentary behaviour and its health effects are not well-known by the students). Some other BCTs, however, involved a more interactive delivery and considered input from the students, as it was thought this could help students to be more engaged with the intervention session. This applied to the three activities within the one-on-one session. For example, students had the chance to verbalise what they thought about the feedback received (e.g., were they surprised by the amount of time they spent sitting?), were invited to think about the potential benefits and disadvantages of changing sedentary behaviour, and were asked about their overall impression of the suggested strategies (including whether they could think of their own strategies).

### Outcome Measures

Outcomes included accelerometer-assessed and self-reported sedentary time, as well as accelerometer-assessed number of steps and prolonged sedentary time (i.e., time spent in sedentary bout durations of ≥30 min and ≥60 min; number of sit-to-stand transitions). In addition, participants completed a sociodemographic questionnaire during the first face-to-face meeting.

The accelerometer used was the activPAL (PAL Technologies, Glasgow, UK), which provides steps and activity counts, as well as inclinometer information used to determine posture. The activPAL is considered the gold standard for the measurement of sedentary behaviour (Kozey-Keadle et al., 2011; Koster et al., 2016) and has demonstrated excellent reliability and validity in measuring sitting, standing, and stepping (Sellers et al., 2016). The device is worn on the midpoint of the anterior aspect of the thigh and is attached to the skin using a hydrogel adhesive pad. The accelerometer can be waterproofed with a small flexible sleeve. Participants received verbal and written instructions to attach the activPAL during the first session and were asked to wear the device on a continuous wearing protocol (i.e., during sleeping and waking hours, including water-based activities). In addition, participants filled in a paper-based daily log collecting data on waking/sleeping hours and wear-related information (e.g., removal periods and reasons).

The self-report tool used to assess sedentary time was the Nightly-Week-U (NWU). The NWU is a validated questionnaire aimed at collecting daily sedentary times of undergraduate students in nine different domains, including work, transport, or socialising (Moulin et al., 2020). Self-reports that prompt participants to examine different areas where they can accumulate sedentary time exhibit more accurate estimates than single-item self-reports (Healy et al., 2011). In addition, a noteworthy element of the NWU is that participants complete the questionnaire at the end of their day (right before retiring to bed), which has been shown to reduce recall bias and increase accuracy compared to weekly self-reported measures (Moulin et al., 2020). The NWU was filled in daily, together with the activPAL log (Supplementary File 3—daily log).

### Process Evaluation Interview

An interview schedule was developed around the three categories included in the MRC process evaluation framework (i.e., implementation, intermediate mechanisms, and context). Areas explored included: strategies used to reduce and break up sedentary behaviour, facilitators and barriers to changing behaviour, perceived mechanisms of action, feedback on the intervention delivery and measurement tools, and role of external factors in influencing sedentary patterns. The schedule consisted of semi-structured, open-ended questions, with additional prompts used if further clarification was needed (Supplementary File 4—interview schedule). For the “intermediate mechanisms” section of the interview, a list of possible mechanisms of action (i.e., processes through which behaviour change occurs) was generated based on the Theory and Technique Tool (Michie et al., 2018, 2021). This online tool consists of a heat map with 74 BCTs (y-axis) and 26 mechanisms of action (x-axis). Each resulting cell uses a colour code to represent the strength of the link between a BCT and a mechanism of action, based on data triangulation from a literature synthesis study (Carey et al., 2019) and an expert consensus study (Connell et al., 2019). Considering the 17 BCTs included in the intervention, 14 mechanisms of action were identified as likely to mediate the effect of the intervention on sedentary behaviour and explain how change occurred. A statement was generated for each mechanism of action (e.g., Beliefs about consequences: “I've changed my sitting patterns over the past week because I'm now aware of the negative consequences of too much sitting”). During the interview, students were asked to rate their agreement with the statements (from 0 to 10), and invited to add additional insights on how the specific mechanism influenced (or not) their sedentary behaviour patterns (Supplementary File 5—mechanism of action questionnaire).

The initial versions for the interview schedule and the mechanisms of action questionnaire were developed by OC and later refined based on feedback from a member of the research team with expertise in qualitative research (IV). Also, the interview was piloted with one University student before the start of the data collection. Minor changes were made to the wording of two questions based on this pilot work.

### Data Analysis

Potential changes in sedentary behaviour patterns and the number of steps from pre- to post-test were examined using paired *t*-tests (normally distributed data) or Wilcoxon tests (non-normally distributed data). The data normality assumption was deemed plausible for all activPAL outcomes, based on statistical (Shapiro-Wilk Test) and graphical (Q-Q plots) procedures, but was rejected for the NWU dimensions (Mishra et al., 2019). The alpha level for significance testing was set as *p* ≤ 0.05 (two-tailed). In addition, effect sizes (hedges' g and rank-biserial correlation) were calculated to describe the magnitude of differences between pre- and post-test, with 0.2, 0.5, and 0.8 indicating a small, medium, and large effects, respectively (Lakens, 2013). All analyses were conducted in SPSS v26.0 (SPSS Inc., NY, USA).

ActivPAL data were exported (EventsXYZ.csv file) and processed following existing recommendations (Edwardson et al., 2017). The activPAL and Excel software packages were used to facilitate the analysis. All events during the self-reported sleeping period were excluded. If not reported (*n* = 2 days), the sleeping period was estimated by visually scanning the time-stamped events file (i.e., identifying cessation and resumption of standing/stepping events during night hours). After removing the sleeping period, the following were summed up for each day and means were calculated from valid days (including separate means for weekdays and weekend): total sedentary time, prolonged sedentary behaviour (sitting/lying bouts of ≥30 min and ≥60 min), number of sit-to-stand transitions, and number of steps. Consistent with previous studies (Edwardson et al., 2017), a day was considered valid if wear time comprised ≥80% of reported waking hours. As with the activPAL data, means for the different dimensions of the NWU questionnaire were calculated for pre- and post-test assessments and inputted in the analyses.

For the process evaluation data, all interviews were audio-recorded and transcribed verbatim by OC. Inductive thematic analysis was applied to identify and organise relevant themes (Braun and Clarke, 2006, 2019). First, participants received a copy of the interview transcript by email and were invited to add information or amendments if they so wished (member checking). Minimal revisions were made by two students. Second, each transcript was coded by OC, with previous transcripts revisited as new codes were identified. Third, codes were grouped into themes/sub-themes and reviewed through rereading full transcripts and coded material, as well as discussion with other members of the research team (IV). An additional deductive step was followed to further analyse the interviews' mechanisms of action results, using direct content analysis (Hsieh and Shannon, 2005). This entailed grouping the themes identified through thematic analysis into the relevant mechanisms of action, as described in the Theory and Technique tool (Michie et al., 2018, 2021). NVivo software was used to facilitate the analysis (QSR International Pty Ltd, version 11). To judge theme relevance, the specific frequency of participants endorsing each theme was considered. Epistemologically, qualitative analyses were situated within an essentialist/realism paradigm (Bhaskar, 2013). For the mechanisms of action questionnaire used during the interview, means for each dimension were calculated.

## Results

### Outcome Evaluation

Nine participants provided informed consent and received the intervention (Table 3). In general, the students provided complete outcome data at both time points (i.e., baseline and post-intervention). For the NWU, 2 days from two different participants were excluded from the analysis due to missing data. For the activPAL, 1 day was excluded from the analysis due to accelerometer malfunction (battery fault). Mean wear time was 98.8% (±3.1). Program retention was 100% and there were no adverse events reported.

**Table 3:** Characteristics of participants in the pilot trial and process evaluation (*n* = 9).

**Variables**	**% (*n*), Mean ± SD**
Gender (females)	56% (5)
Age	22 ± 2.32
**Year of undergraduate study**	
1st year	22% (2)
2nd year	33% (3)
3rd year	45% (4)
**Major subject of study**	
Business economics	11% (1)
Finance	22% (2)
Law	22% (2)
Primary education	11% (1)
Mixed courses (e.g., finance and accounting)	33% (3)
**Race/Ethnicity**	
White	89% (8)
Pacific Islander	11% (1)
**Employment status**	
Student (only)	67% (6)
Student with part-time job	33% (3)
**Residency**	
On-campus	11% (1)
Off-campus	89% (8)

#### Changes in Accelerometer-Derived Sedentary Time, Standing and Moving

Table 4 shows the descriptive statistics for each of the activPAL outcomes, including a pre-post comparison (paired *t*-tests). From baseline to post-intervention, there was a statistically significant reduction in total and prolonged sedentary time during weekend days (mean baseline [SD], mean change, *p*-value—[a] sedentary time: 11.06 h/d [1.6], −1.65 h/d, *p* = 0.005; [b] Time spent in sedentary bouts >30 min: 7.2 h/d [2.1], −1.65 h/d, *p* = 0.007; [c] Time spent in sedentary bouts >60 min: 4.25 h/d [2.36], −2.03 h/d, *p* = 0.002). In addition, there was a statistically significant increase in time spent standing and stepping during weekend days (standing time: 2.86 h/d [1.36], 1.1 h/d, *p* = 0.019; stepping time: 1.28 h/d [0.33], 0.55 h/d, *p* = 0.003; number of steps: 5585.11 [1554.06], 2513.33, *p* = 0.004). There were no statistically significant changes in sedentary time, standing, or moving across the whole 6 day period or during weekdays.

**Table 4:** Descriptive statistics and pre-post comparison (paired *t*-tests) for the activPAL outcomes (*n* = 9).

**Variables**	**Pre^a^**	**Post^a^**	**Mean difference**	***p*-value^b^**	**Effect size^c^**
**Total (6 days)**					
Sitting time	10.62 (0.99)	10.17 (1.35)	−0.45	0.12	−0.58
Standing time	3.10 (0.77)	3.38 (1.20)	0.28	0.22	0.44
Stepping time	1.49 (0.36)	1.66 (0.4)	0.17	0.125	0.57
Time in sitting bouts >30 min	6.58 (1.64)	6.10 (2.08)	−0.48	0.285	−0.38
Time in sitting bouts >60 min	3.50 (1.38)	3.00 (1.7)	−0.50	0.274	−0.39
Sit-to-stand transitions	42.79 (9.20)	43.27 (8.28)	0.48	0.863	0.05
Step count	6962.4 (1898.89)	7615.92 (2173.10)	653.51	0.189	0.47
**Weekdays**					
Sitting time	10.39 (0.93)	10.55 (1.45)	0.15	0.722	0.12
Standing time	3.2 (0.7)	3.09 (1.26)	−0.11	0.734	−0.11
Stepping time	1.61 (0.42)	1.57 (0.46)	−0.03	0.82	−0.07
Time in sitting bouts >30 min	6.26 (1.74)	6.38 (2.15)	0.13	0.855	0.06
Time in sitting bouts >60 min	3.13 (1.15)	3.39 (1.61)	0.26	0.659	0.15
Sit-to-stand transitions	43.38 (10.69)	46.08 (9.97)	2.69	0.517	0.22
Step count	7693.38 (2258.18)	7374.66 (2401.63)	−318.72	0.618	−0.17
**Weekend**					
Sitting time	11.06 (1.6)	9.41 (1.99)	−1.65	**0.005**	−1.27
Standing time	2.86 (1.36)	3.96 (1.71)	1.10	**0.019**	0.97
Stepping time	1.28 (0.33)	1.83 (0.51)	0.55	**0.003**	1.44
Time in sitting bouts >30 min	7.2 (2.1)	5.55 (2.66)	−1.65	**0.007**	−1.18
Time in sitting bouts >60 min	4.25 (2.36)	2.22 (2.12)	−2.03	**0.002**	−1.44
Sit-to-stand transitions	41.72 (7.67)	37.66 (9.93)	−4.05	0.092	−0.63
Step count	5585.11 (1554.06)	8098.44 (2529.31)	2513.33	**0.004**	1.32

a *Mean hours/steps per day (standard deviation)*.

b *Bold text indicates p < 0.05 for paired t-test*.

c *Effect size = Hedges' g (Grissom and Kim, 2005)*.

#### Changes in Self-Reported Sedentary Time

Table 5 shows the descriptive statistics for each of the NWU dimensions, including a pre-post comparison (related samples Wilcoxon Signed Rank Test). From baseline to post-intervention, there was a statistically significant reduction in total self-reported sedentary time across the whole 6 day period and during weekend days (median baseline hours per day [IQR], median change, *p*-value—[a] sedentary time: 10.31 h/d [2.14], −1.3 h/d, *p* = 0.021; [b] sedentary time during weekend days: 10.69 h/d [3.25], −1.92 h/d, *p* = 0.021). In addition, there was a statistically significant increase in time spent studying during weekdays (1.73 h/d [1.75], 1.37 h/d, *p* = 0.028). There were no statistically significant changes in self-reported sedentary time for the rest of the NWU dimensions.

**Table 5:** Descriptive statistics and pre-post comparison (related samples Wilcoxon Signed Rank Test) for each of the Nightly-Week-U dimensions (*n* = 9).

**Variables**	**Pre^a^**	**Post^a^**	**Median difference**	***p*-value^b^**	**Effect size^c^**
**Total (6 days)**					
Sitting time	10.31 (2.14)	9.01 (2.06)	−1.30	**0.021**	−0.54
Sitting for study	1.81 (1.62)	2.30 (2.17)	0.49	0.678	−0.10
Sitting for work	0.21 (0.89)	0.25 (0.53)	0.04	0.345	0.22
Sitting for transport	0.73 (0.5)	0.74 (0.7)	0.01	0.953	0.01
TV viewing	0.81 (2.31)	0.50 (1.79)	−0.31	0.401	−0.20
Computer use	1.86 (1.53)	1.73 (2.45)	−0.13	0.767	−0.07
Sitting for leisure reading	0.04 (0.34)	0 (0.56)	−0.04	0.917	−0.02
Sitting for eating	1.15 (0.79)	0.78 (0.61)	−0.38	0.441	−0.18
Sitting for socialising	0.8 (0.95)	0.78 (1.14)	−0.02	0.484	−0.16
Sitting for other purposes	1.14 (1.23)	1.10 (0.74)	−0.04	0.214	−0.29
**Weekdays**					
Sitting time	10.14 (1.97)	9.13 (2.19)	−1.01	0.139	−0.35
Sitting for study	1.73 (1.75)	3.10 (2.16)	1.37	**0.028**	0.52
Sitting for work	0.14 (1.34)	0.16 (0.43)	0.02	0.345	0.22
Sitting for transport	0.75 (0.63)	0.38 (0.86)	−0.37	0.953	−0.01
TV viewing	1.21 (2.31)	0.23 (1.81)	−0.98	0.176	−0.32
Computer use	2.09 (1.34)	1.20 (1.94)	−0.89	0.109	−0.38
Sitting for leisure reading	0.06 (0.39)	0 (0.12)	−0.06	0.345	−0.22
Sitting for eating	1.15 (0.65)	0.71 (0.76)	−0.44	0.314	−0.24
Sitting for socialising	0.85 (1.41)	0.34 (1.56)	−0.5	0.779	−0.07
Sitting for other purposes	0.68 (1.76)	0.81 (1.18)	0.12	0.678	0.1
**Weekend**					
Sitting time	10.69 (3.25)	8.76 (4.48)	−1.92	**0.021**	−0.54
Sitting for study	1.52 (3.3)	1.41 (2.61)	−0.11	0.401	−0.2
Sitting for work	0 (0.37)	0 (0.66)	0	1.000	0
Sitting for transport	0.52 (0.37)	0.7 (0.9)	0.17	0.594	0.13
TV viewing	0.51 (2.68)	1.05 (2.21)	0.54	0.889	0.03
Computer use	2.06 (3.04)	1.45 (2.16)	−0.62	0.515	−0.15
Sitting for leisure reading	0 (0.21)	0 (0.46)	0	1.000	0.00
Sitting for eating	1.04 (1.11)	0.87 (0.34)	−0.18	0.214	−0.29
Sitting for socialising	1.17 (1.76)	0.78 (1.34)	−0.39	0.499	−0.16
Sitting for other purposes	1.55 (2.43)	1.39 (1.67)	−0.17	0.263	−0.26

a *Median hours/steps per day (interquartile range)*.

b *Bold text indicates p < 0.05 for related samples Wilcoxon Signed Rank Test*.

c *Effect size = Rank-biserial correlation (Rosenthal et al., 1994)*.

## Results

### Process Evaluation

Interviews ranged from 23 to 38 min in duration, with a mean of 30.8 min per interview (± 4.67). Overall, four main themes where identified: “implementation,” “context,” “mechanisms of action,” and “behaviour change experience.” These themes are presented below, including relevant first and second level sub-themes (see Table 6 for a complete list of themes, together with example quotes from participants and frequency counts). In addition, the summary results from the mechanism of action questionnaire are provided in Table 7.

**Table 6:** Themes elicited from process evaluation interviews with University students (*n* = 9).

**Themes**	**Category**	**Subcategory**	**Exemplar statement**	**Frequency across interviews (*n* = 9)**
Implementation	Assessment	Wearing the activPAL was comfortable	“*It didn't bother me at all. After a few hours you just forget it is there.”*	8
		Data collection reminders via text messages were helpful	“*The reminder to collect the data at the end of the day was really good, just to remember about it.”*	7
		Too many wear days (activPAL)	“*Towards the end of the second week I got a bit fed up, especially at night, as I sleep face down.”*	2
	Intervention	The intervention session was helpful and clearly delivered.	“*I think everything was really clear. I liked the visuals, that really helped me, and also the definitions for different physical activities. It was very informative and it made me think a lot about how much sitting really affects me.”*	8
		I didn't use any of the apps suggested	“*I did look at the apps, I just didn't get around to using them myself.”*	8
		The intervention text messages were helpful	“*I thought the text messages were really great. With some of the apps I've tried, they were just like ‘get up and get a drink of water now, bla bla bla’. But with the text messages I thought that was better for me because there were reasons, suggestions, etc all different types of reminders, more complex.”*	7
		Poster was an effective visual cue	“*I had the poster on my desk and when I was studying I looked at it and I was like ‘think outside the chair’* (poster phrase)*, that was really good, as a cue.”*	4
		The intervention text messages were irrelevant	“*It wasn't helpful for me personally. I'm not on my phone, I don't keep my phone with me that much. Most people are attached to their phones. I'm not one of them.”*	2
Context	University workload	Higher levels of total and prolonged sedentary behaviour during the exam period	“*Towards the end of the semester, when I start doing exam revision, I probably sit down more to focus, because I can't… otherwise my mind wanders a bit too much. So, yes, the closer to exam time, I probably sit a lot longer.”*	8
	External influences during the study period	Pre and post periods were comparable	“*In general yes. That was the only out of the norm thing. But generally my routine didn't change at all, so I studied the same, worked the same, etc.”*	8
	Weather	Higher levels of total and prolonged sedentary behaviour during winter/summer	“*In winter, when it's cold, I'm more likely to grab a blanket and wrap up. In summer, I'd be sitting down as well, because it's too hot to do anything. So the weather does affect me.”*	5
	Work	Higher levels of total and prolonged sedentary behaviour during days off	“*For me, because I do work, I do stand a lot when I work. Then I'd tend to sit a lot when I'm at home. I just sit a lot.”*	2
Mechanisms of action^a^	Knowledge	Increased knowledge about sedentary behaviour	“*It has influenced me yes. What I've learnt about sitting behaviour, and why it matters. I think the access to the information that you gave me has definitely opened up my mind.”*	8
	Attitude towards the behaviour	Negative attitude towards *too much* sitting	“*Yeah, definitely. As I said, the other day I just got fed up seeing how much I was actually sitting down. I got sick and tired of watching TV every day. And I'm like ‘no, get out’.”*	7
		Sedentary behaviour is not inherently bad	“*I didn't really see it… I don't have a negative attitude towards too much sitting. It can help me to get my degree, for example.”*	2
	Feedback processes	Feedback was eye-opening	“*I was just shocked by that day I spent 20 hours sitting. I think seeing the data there really was like ‘okay, that's just the facts, I have to change it, I can't argue it’. It definitely did motivate me.”*	8
	Motivation	Increased motivation	“*I feel I'm more motivated, not just for* (reducing) *sitting but also to be more active in general, like walking more. I catch an Uber for everything…”*	7
	Belief about consequences	Reducing total and prolonged sedentary behaviour is good for your physical health	“*I think even… I've had a lot of back pain in the past, and that has felt better this week because I've gone out walking, I've spent more time standing up.”*	8
		Reducing total and prolonged sedentary behaviour is good for your mental health	“*For me it was definitely the mental aspect. It kind of refreshes you. If you do something for too long and then once you stand up, I feel it kind of refreshes my mind a little.”*	6
		Breaking up sedentary behaviour helps you to be more organised	“*It has helped me to organise my time more efficiently, by breaking up my day. Usually I'm so disorganised.”*	2
	Behavioural cueing	Text messages as effective prompts/cues	“*One of the really good things were the text messages, as a reminders. It helped me, I think if I didn't have it would have been a lot harder* (change behaviour)*.”*	7
		Visual cues as effective triggers	“*Looking at the poster was a good reminder, especially when I was tired and started looking away from the screen.”*	*4*
	Intentions	Conscious decision to reduce total and prolonged sedentary behaviour	“*I feel I was thinking about it a lot this week. Before I was not actively trying to make changes in this area, I'd be mindlessly sitting.”*	6
	Behavioural regulation	Goals to reduce total and prolonged sedentary behaviour	“*One of my goals was to go for a walk and one was to do the dishes, and that sort of thing… so having goals was good. I'm a bit of an achievement-hunter, so I really wanted to achieve the goal. I think that was really helpful.”*	4
		Self-monitoring of sedentary behaviour	“*I kind of tracked my behaviour when I was studying, with the computer clock. For example, ‘I wanna do three hours and I divide it in blocks of 30-45 minutes’.”*	5
	Skills	Develop new skills	“*Yes, I've developed new skills, based on some of the strategies we discussed to reduce and break up my sitting time.”*	5
		Use existing skills	“*I'd say no. Because I wasn't doing things that I was not doing before, but just maybe more often.”*	4
	Social/professional role and identity	Part of the student role	“*Yes, cause it's mainly when I'm more conscious of breaking up my sitting, when I'm studying.”*	5
		Not part of the student role	“*A little bit. I don't see it as a huge part of my student role. Studying, completing assignments, absolutely, but this one, not sure…”*	4
	Reinforcement	I don't bribe myself	“*I don't know. I didn't really use prizes or anything like that.”*	7
		Use of snack breaks	“*I guess I could call it… when I have a break to stand up I'm having a snack, and drink water or* (have) *something to eat.”*	2
	Subjective norms	Reducing and breaking up sedentary behaviour is not emphasised in the University setting	“*Not really, I definitely don't think it's even acknowledged at the uni. There's no real focus on activity in the courses I'm in, or any of the people I do the course with. There is not a focus into a healthy lifestyle.”*	8
	Social influences	No external influences	“*Not really. It was my individual behaviour. My parents were aware I was participating in the study, but they didn't influence me.”*	7
		I've tried to convince others to reduce their sedentary behaviour	“*I haven't had a lot of social support… it was more me trying to help him* (partner).”	5
		Family members promote change	“*Yes, my dad, he nudges, he's like ‘get up, do this, do that’, so I think one of the days I was just sitting down reading and he came and annoyed me to go and tidy up the yard, so he pretty much bugged me to get up. They knew I was going to do this as well* (participating in the study)*.”*	2
	Self-image	Not long enough to change one's conception of oneself	“*Not so much in a week. If I continue over a month or so I'll definitely… I feel I've been standing a fair bit more though.”*	8
Behaviour change process	Strategies	Perform tasks standing or moving rather than sitting	“*The activities I could do standing, I just did it, like talking on the phone or something like that.”*	7
		More household jobs	“*Yeah, doing a lot more households jobs. I was more motivated to break up sitting by doing something else in between sitting, like study for a little bit and then get up, fold the washing, or actually cook a meal or something like that, and then go and sit down.”*	4
		Use of visual cues	“*I put sticky notes on my laptop to just remind me to break up sitting. I think I'm probably more like a visual person.”*	4
		Take the long way	“*I did the ‘take the long way’ thing* (suggested strategy)*, for example this morning I took a diversion to come here and meet you, to make it longer.”*	3
	Barriers	Easy to forget about it	“*I'm like one person that when I'm studying I kind of forget* (to reduce and break up sitting)*, or if I'm on my phone… I just don't see the time.”*	7
		Goal conflict with studying	“*It was hard to maintain* (behaviour change)*, with external factors like studying, classes, etc.”*	6
		Social norm to sit	“*My family came, so I sat a lot for socialising. I didn't want to be rude and just stand up during the conversation. That was another factor, just being with your friends or family… ‘oh I need to stand up’, and they say ‘why are you standing up? Are you okay?’.”*	3
		Difficult to ‘break’ a habit	“*Nothing preventing me* (from reducing and breaking up sedentary behaviour)*, just the habit I guess. It's hard after only six days. I think it takes a little bit longer to create a habit, so if it was a bit more prolonged I definitely think you see more changes in my patterns.”*	2
	How sedentary behaviour was substituted	A combination of standing and walking	“*I did go for a few more walks this week, but yeah, when I had the breaks I was mostly standing, not really walking that much.”*	3
		Mainly walking	“*When I wasn't sitting I was probably just walking around, maybe do something in the house like clean up or stuff like that.”*	3
		Mainly standing	“*I was replacing it* (sitting) *mainly with standing. The activities I could do standing, I just did it. I tried studying while standing a little bit as well.”*	3
	Occupational vs non-occupational	Recreational easier	“*Recreational is easier, because if I'm listening to a lecture I kind of have to sit there and listen to it. With leisure activities I can make choices* (to reduce and break up sitting). *For example, going for a walk with friends.”*	7
		Studying easier	“*Studying was easier, because I had the timer. ‘Times is up, time to move away’, as opposed to looking at something else. Because I'm not really thinking about sitting when I'm watching TV. I didn't make a conscious effort to get up.”*	1
	Future behaviour	I'll continue reducing and breaking up my sedentary time	“*I'm going to take what I've learnt from this, cause really it's not that hard just to stand when you don't have to be sitting. So I think I'll continue and find more ways to reduce my sitting in total.”*	9

a *Unlike the rest of themes, the “mechanism of action” categories are based on pre-specified constructs, as described in the Theory and Technique Tool (Michie et al., 2018)*.

**Table 7:** Mean score (0–10) for the 14 mechanisms of action statements explored in the process evaluation interviews with University students (*n* = 9).

**Mechanism of action^a^**	**Mean score**	**Standard deviation**
Feedback processes	9.22	0.66
Motivation	8.33	1.41
Intentions	8	1.58
Belief about consequences	7.78	1.39
Knowledge	7.67	2.12
Behavioural cueing	6.89	2.20
Attitude towards behaviour	6.67	2.12
Social/professional role	5.33	2.59
Behavioural regulation	5.29	1.39
Skills	5	2.64
Self-image	3.67	2.73
Reinforcement	3.33	2.17
Social influences	3.22	1.48
Subjective norms	2.78	0.97

a ***Feedback processes**: Processes through which current behaviour is compared against a particular standard. **Motivation**: Processes relating to the impetus that gives purpose or direction to behaviour and operates at a conscious or unconscious level. **Intentions**: A conscious decision to perform a behaviour or a resolve to act in a certain way. **Belief about consequences**: Beliefs about the consequences of a behaviour (i.e., perceptions about what will be achieved and/or lost by undertaking a behaviour, as well as the probability that a behaviour will lead to a specific outcome). **Knowledge**: An awareness of the existence of something. **Behavioural cueing**: Processes by which behaviour is triggered from either the external environment, the performance of another behaviour, or from ideas appearing in consciousness. **Attitude towards the behaviour**: The general evaluations of the behaviour on a scale ranging from negative to positive. **Social/professional role and identity**: A coherent set of behaviours and displayed personal qualities of an individual in a social or work setting. **Behavioural regulation**: Behavioural, cognitive, and/or emotional skills for managing or changing behaviour. **Skills**: An ability or proficiency acquired through practise. **Self-image**: One's conception and evaluation of oneself, including psychological and physical characteristics, qualities, and skills. **Reinforcement**: Processes by which the frequency or probability of a response is increased through a dependent relationship or contingency with a stimulus or circumstance. **Social influences**: Those interpersonal processes that can cause oneself to change one's thoughts, feelings, or behaviours. **Subjective norms**: One's perceptions of what most other people within a social group believe and do*.

#### Theme 1: Implementation

Regarding the data collection, interviews with students showed that they were generally positive about the activPAL and felt that it did not affect their daily activities. However, the number of wear days was identified as a barrier to trial participation by some participants. There was consensus among the students that the text message reminders were beneficial for the completeness of the daily logs and NWU questionnaire.

Regarding the intervention, there was a clear positive response towards the one-on-one session, which was described as highly informative and useful to achieve behaviour change. The intervention text messages were also well-received, although two students reported that they were not relevant to them because of limited smartphone use. Participants had very few ideas or recommendations to improve the intervention content or delivery, but one student suggested the information provided should be more tailored to University students. Approximately half of the students used the provided poster as a visual cue to reduce and break up sedentary behaviour, while suggested apps were generally not used.

#### Theme 2: Context

Students identified several environmental factors influencing their sedentary behaviour patterns. University workload was highlighted as a potential source of variation, with most participants stating that they are less likely to reduce and break their sedentary time as workload increases. The weather was also identified as a relevant factor. Participants linked extreme weather conditions (i.e., hot in summer, cold in winter) to higher levels of sedentary behaviour. In addition, two out of the three students who work part-time (on top of their studies) claimed that they tend to accumulate more sitting during days off work (non-University related), reflecting some sort of compensation behaviour. Finally, a majority of students expressed the view that both trial weeks (pre and post) were comparable, in terms of external influences to their sedentary behaviour.

#### Theme 3: Behaviour Change Experience

The key strategies used by the students to reduce and break up sedentary behaviour centred on performing daily activities while standing or moving (rather than sitting), doing more household work, and using visual cues (poster or post-its). Moreover, some students tried to incorporate active time into their daily commutes (e.g., parking the car further away). Participants also reported a series of barriers that made behaviour change difficult. These included competing demands between reducing sedentary time and studying (i.e., goal conflict), as well as difficulties remembering to reduce or break up sedentary behaviour while performing other activities. Additionally, most students' responses reflected that it is easier to change sedentary behaviour patterns during recreational, non-University related activities.

While all participants claimed to have reduced the amount of time spent sedentary during the post-intervention period, there were differences in how sedentary behaviour was displaced. Some participants substituted sedentary behaviour mainly with walking, whereas others replaced sedentary behaviour with standing or a combination of walking and standing activities. All students stated that they would actively try to be less sedentary in the future.

#### Theme 4: Mechanisms of Action

For most participants, the knowledge learnt from the intervention had a powerful impact on their behaviour change process. The one-on-one session and supporting text messages were generally viewed as successful in raising awareness of the physical and mental health consequences of too much sitting and for increasing motivation to make a change. As a result, most answers reflected a negative attitude towards excessive sedentary behaviour, developed as a result of trial participation, and an intention to introduce changes.

The students highlighted the activPAL feedback provided during the one-on-one session and the text messages as two intervention components that were particularly helpful in their quest to reduce and break up sedentary behaviour. To a lesser extent, students also attributed their behaviour change success to the use of self-regulation strategies (i.e., goal setting and self-monitoring). There were conflicting views, however, on whether the intervention assisted the students in developing new skills to reduce and break up their sedentary behaviour. Some students did not consider that they have learnt new skills but rather used existing strategies more often. Similarly, students held mixed views as to whether reducing and breaking up sedentary time is part of their student role/identity.

Other mechanisms of action explored during the interviews (i.e., reinforcement, subjective norms, social influences, and self-image) did not seem to have substantially contributed to the students' behaviour change processes. For example, only two students reported having used incentives to reinforce behaviour change efforts. Similarly, most students did not mention any social influence that had positively contributed to reducing and breaking up their sedentary behaviour, although many claimed that they have tried to ‘convince’ their family and friends to move more and sit less.

#### Mechanism of Action Questionnaire

As shown in Table 7, the mean scores for the different mechanisms of action statements (i.e., reasons for change) ranged from 2.8 to 9.2, on a scale of 0 to 10 (with 0 meaning that the specific statement did not apply to the student's behaviour change process at all, and 10 meaning that it completely reflected the student's reason for change). Statements referring to feedback processes, motivation, intentions, beliefs about consequences, knowledge, behavioural cueing, and attitude towards the behaviour received a mean score above five (scale's midpoint). Statements referring to social/professional role, behavioural regulation, and skills received a mean score of (or close to) five. Finally, statements referring to self-image, reinforcement, social influences, and subjective norms received a mean score below five.

## Discussion

The purpose of this study was to assess the feasibility and preliminary, short-term effects of a BCW-informed intervention aimed at reducing total and prolonged sedentary behaviour in University students. Among our small sample of University students, both accelerometer and self-reported findings suggest that a one-on-one session, together with daily text messages, might encourage students to reduce sedentary behaviour and increase standing and stepping during weekend days. Effect sizes were large for the accelerometer data (activPAL), and small-to-medium for the self-report data (NWU questionnaire). However, there were no significant changes in sedentary behaviour, standing, or stepping across the whole 6 day period or during weekdays.

While limited statistical power may have made it difficult to detect statistically significant changes, results suggest the intervention had different effects depending on the day of the week. Based on the process evaluation results, we interpret this might be because of the type of activities predominantly performed during weekend and weekdays (i.e., recreational and occupational, respectively). Students reported finding it easier to change their sedentary behaviour patterns during leisure activities, rather than during University-related tasks. This is due to common concerns about the negative impact that reducing and breaking up occupational sitting might have on performance, similar to those reported in previous qualitative studies with University students (Castro et al., 2020b) and office workers (Cole et al., 2015; MacDonald et al., 2018).

Considering the students found it particularly difficult to reduce and break up their sedentary time during occupational activities, another factor that might have contributed to the lack of change in sedentary behaviour during week days is the reported increase in time spent carrying out academic activities for that period. From baseline to post-intervention, “studying” during weekdays was the only self-reported NWU dimension that experienced a significant change. Even if students also undertake academic activities at home during the weekend, it is plausible that these allow for more active choices, compared to attending lectures or studying in the library on weekdays (where students might not have the option to reduce and break up their sedentary behaviour, or might find it more difficult). The latter may include social normative influences that reduces the chances of students making changes (Pachu et al., 2020).

Taken together, these findings suggest that a more complex intervention, involving relevant staff from the University setting and wider environmental changes, are likely to be needed to maximise behaviour change (especially for occupational sedentary behaviour during weekdays). One potential avenue for change is University lecturers, who are in a unique position to highlight the importance of reducing and breaking sedentary behaviour and promote social acceptability for changing behaviour (e.g., implementing active breaks during their lectures). Yet, the interviews reflected that this is not currently being emphasised by lecturers, student wellness advisors, or any other University staff members. Future research should examine how sedentary reduction interventions could be conducted utilising these staff and their potential to be facilitators of behaviour change. In addition, the introduction of sit-to-stand desks in University classrooms and libraries is an effective approach to reducing sedentary behaviour in University students (Tardif et al., 2018, Jerome et al., 2017). These desks allow individuals to displace large volumes of sitting to standing, while generally causing little to no disruption in productivity and work routines (Karakolis and Callaghan, 2014; Ojo et al., 2018). However, it should be noted that incorporating such environmental opportunities are not always feasible given the increased intervention cost.

Process evaluation results indicated that the intervention protocol and its assessment is feasible and acceptable. Some students felt, however, that the number of days for wearing the activPAL was too long. This could hamper student retention, particularly if further assessments are planned beyond pre- and post-test measurements (e.g., follow-up). One option could be reducing the number of wear days per assessment. A recent study showed that activPAL data from 5 wear days provide precise estimates of weekly activity behaviour in adults, as long as at least 1 weekend day is included (Aguilar-Farias et al., 2019).

Overall, students gave positive feedback for, and engaged with, the majority of intervention components, except for the smartphone apps list. Given the high levels of smartphone penetration and use among young adults in Australia (Oviedo-Trespalacios et al., 2019), we were surprised that the students did not generally download any of the suggested apps to facilitate sedentary behaviour change. It is likely that the apps were not used because students were already receiving automatic reminders and information regarding sedentary behaviour via text messages, which is similar to what most freely available apps targeting sedentary behaviour offer (using “push notifications” instead of text messages). Some students mentioned that they were going to re-examine the apps list once the scheduled text messages stopped, as an alternative way to receive prompts. The use of text messages has proved effective in previous studies targeting health behaviour change in University students and constitutes a promising, low-cost intervention approach (Obermayer et al., 2004; Head et al., 2013).

An important component of the process evaluation consisted of exploring the intervention's mechanisms of action, based on the BCTs implemented. Relevant mechanisms identified through the interviews, and supported by the results from the mechanism of action questionnaire, included: feedback processes, motivation, intentions, beliefs about consequences, knowledge, behavioural cueing, and attitude towards the behaviour. Most of these mechanisms refer to the “reflective motivation” and “psychological capability” constructs within the COM-B model (Michie et al., 2011). That is, the one-on-one session and complementary information appeared to increase awareness of the health consequences of excessive sedentary behaviour, and thus provided motivation for the students to make changes to the amount of time they spend sedentary. In addition, results from the process evaluation showed that automatic mechanisms also play an important role in reducing sedentary behaviour (“automatic motivation” within the COM-B model). The personalised feedback provided on baseline sedentary behaviour (feedback processes) and the regular text-messages (behavioural cueing) were two intervention components that the students identified as most helpful to achieve the desired change. A common element of these two strategies is that they are based on bringing habitual behaviour into conscious awareness. Given that sedentary behaviour is mostly habitual (i.e., it involves little cognitive engagement and is driven by automatic responses), specific strategies targeting unintentional and habit-like behaviour are needed to better control sedentary time (Compernolle et al., 2019). Feedback/monitoring of behaviour and behavioural cueing are thus two useful approaches to disrupt sedentary behaviour and should be considered in future interventions, together with strategies targeting reflective motivation (e.g., information on health effects).

Having a preliminary understanding of how the intervention works is desirable as it can allow a more detailed analysis during subsequent process evaluations (e.g., using standardised questionnaires). In addition, it can be used to further optimise the intervention. For example, while behaviour change did not seem to be enhanced by social support in our intervention, available research indicates that the health choices of adolescents and young adults are greatly influenced by peers (Yeager et al., 2018). Moreover, our sample of students cited social norms to sit as a barrier to reduce and break up their sedentary behaviour, similar to previous studies with office workers (Mansfield et al., 2018). Based on the above, decisions should be made as to whether to modify or introduce new elements in the intervention to address social support/social norms more directly. For example, this could be done by delivering part of the intervention through group sessions, setting up “active” study groups or a buddy system.

By using the NWU questionnaire, we were able to better understand how the participants spent their sedentary time during the trial period. The dominant sedentary behaviour subdomains in our sample were screen time and academic activities. This is consistent with the results from our recent meta-analysis (Castro et al., 2020a) and suggests that future interventions targeting sedentary behaviour reduction in University students should pay close attention to these two behavioural contexts. Because different sedentary behaviours might be influenced by different factors, targeting specific subdomains or contexts might help intervention developers to identify more precisely what the sources of implementation problems are, thus increasing the likelihood for the intervention to be effective (Michie et al., 2008).

An important element of our intervention consisted of arranging a one-on-one session between the participant and the researcher, where most of the BCTs forming the intervention were delivered. It is worth noting that a rich literature exists on individual, person-centred, counselling approaches that have been shown to be effective in helping people change health-related behaviours (e.g., motivational interviewing; Miller and Rollnick, 2012). While the underlying assumptions and theoretical underpinnings of person-centred counselling and the BCW are somewhat different, we consider there is room for future research investigating how to integrate both approaches. For example, motivational interviewing specifically targets the motivational aspects of change, and in that sense it should be compatible with the motivational component of COM-B. In addition, motivational interviewing places especial emphasis on the vocabulary for change and type of communication used during the individual sessions (i.e., the style of delivery), something that is not appropriately covered within the BCW (which focusses primarily on developing intervention content). The role of using person-centred techniques and communication principles in the delivery of one-on-one behaviour change interventions has not been studied within the context of BCW-framed interventions, however, and deserves to be investigated in the future. Research exploring how to integrate both approaches might help intervention developers incorporate the added benefits of using the BCW (e.g., comprehensiveness, clear and systematic path to developing intervention content), while also taking into account a range of issues that have been proved to be relevant when helping people to change and that were not fully considered in our intervention (e.g., motivational interviewing type of communication, support of basic psychological needs, tailoring of BCTs depending on the participant's stage of change).

Last, a distinctive feature of our intervention is that we aimed to both reduce and break up sedentary behaviour. That is, we were not only interested in helping students to reduce their overall sedentary behaviour levels but also interrupt long periods of sedentary time more often. While these two behavioural targets are grounded in current public health guidelines (e.g., Department of Health, 2014; Department of Health, Physical Activity, Health Improvement and Protection, 2019), some authors have recently questioned the inclusion of sedentary breaks in the evidence-based guidelines, arguing that the studies supporting the beneficial health effects of breaking up sedentary behaviour often present mixed findings and are limited to small samples (Stamatakis et al., 2019). Another disputed topic refers to whether standing is enough to attenuate the negative health effects of total and prolonged sedentary behaviour, as there is only a small difference in energy expenditure between sitting and standing (Bailey and Locke, 2015; Van der Ploeg and Hillsdon, 2017).

Sedentary behaviour is a relatively new area of research within the physical activity and public health field and, consequently, a number of issues remain to be investigated (Biddle et al., 2019). With research on sedentary behaviour growing rapidly, researchers and practitioners should pay close attention to the new advancements in the field and tailor their behavioural targets accordingly. As with physical activity guidelines, recommendations on sedentary behaviour are likely to become more sophisticated over time. In light of the current state of the evidence, we grounded our intervention in the message “sit less, move more, more often,” highlighting that standing is a good start, but the additional movement of any intensity will support stronger health effects in University students. Apart from physical health outcomes, however, future intervention developers should also consider the emerging evidence on sedentary behaviour and psychological outcomes (De Cocker et al., 2020). For example, standing seems to play a positive role in increasing alertness and boost productivity (Biddle et al., 2020), which is particularly relevant to University students.

### Study Strengths and Limitations

Strengths of this study include the use of an evidence- and theory-based framework to develop the intervention, the incorporation of a process evaluation informed by the MRC guidelines, and the assessment of sedentary behaviour by both accelerometer and self-report methods. Limitations are the lack of control condition, the small sample size, and the short-term duration of the study. Although the purpose of the study was to conduct a feasibility investigation (not a thorough evaluation of the intervention's effectiveness), a larger sample size was initially planned, including a control group and an additional follow-up assessment. However, we had to cancel further student recruitment due to the Covid-19 restrictions. For future research evaluating effectiveness, an adequately powered sample of participants with one or more follow-up assessments are needed to ascertain the reliability and sustainability of the behavioural changes observed. In addition, adding a control condition is particularly important, because the students identified several environmental factors influencing their sedentary behaviour patterns over time (e.g., weather, University workload). Last, adequate procedures for establishing data saturation should be implemented in future process evaluation interviews (e.g., Lowe et al., 2018).

## Conclusions

Our findings suggest that a brief, BCW-informed intervention (composed of a single one-on-one session and automated daily text messages) may help University students to reduce sedentary behaviour and enhance movement during weekend days. Based on the process evaluation results, we propose that the intervention effects occurred through changes in the student's reflective and automatic motivation regarding sedentary behaviour. We discuss different strategies that could be added to the current intervention to maximise its potential for reducing and breaking up sedentary behaviour, such as establishing a collaboration with University staff, introducing sit-to-stand desks, and/or facilitating social support. Overall, the intervention's implementation and evaluation were feasible and acceptable to the students. A larger, randomised controlled trial with follow-up assessments is warranted to appropriately evaluate intervention effectiveness.

## Data Availability Statement

The raw data supporting the conclusions of this article will be made available by the authors, without undue reservation.

## Ethics Statement

The studies involving human participants were reviewed and approved by University of Southern Queensland's (USQ) Human Research Ethics Committee (Ethical approval No. H19REA314). The patients/participants provided their written informed consent to participate in this study.

## Author Contributions

OC, IV, JB, and SB contributed to the conception and design of the study. OC collected the data, performed the quantitative and qualitative analyses, with input from IV (qualitative analysis), and developed the first draft of the paper. JB, IV, and SB assisted with the interpretation of findings. All authors contributed to the drafting and revision of the final article and approved the final submitted version of the manuscript.

## Conflict of Interest

The authors declare that the research was conducted in the absence of any commercial or financial relationships that could be construed as a potential conflict of interest.
