# Supplementary material for: Feasibility of Reducing and Breaking Up University Students' Sedentary Behaviour: Pilot Trial and Process Evaluation
Source: Front Psychol. 2021 Jun 10;12:661994. doi: 10.3389/fpsyg.2021.661994 (PMC8222591; doi:10.3389/fpsyg.2021.661994)
Supplement: Supplementary file 1 [file Table_1.DOCX]

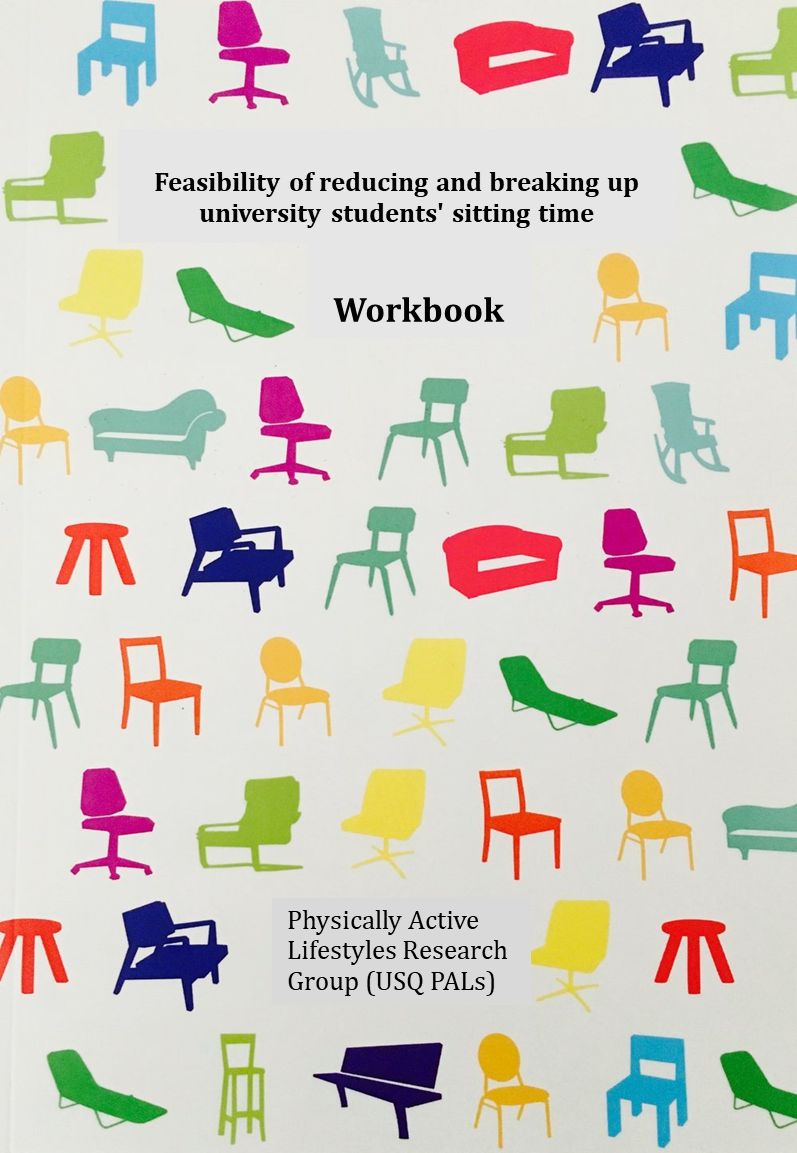

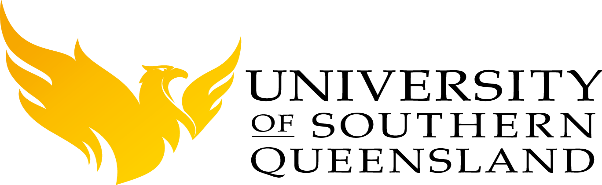


| 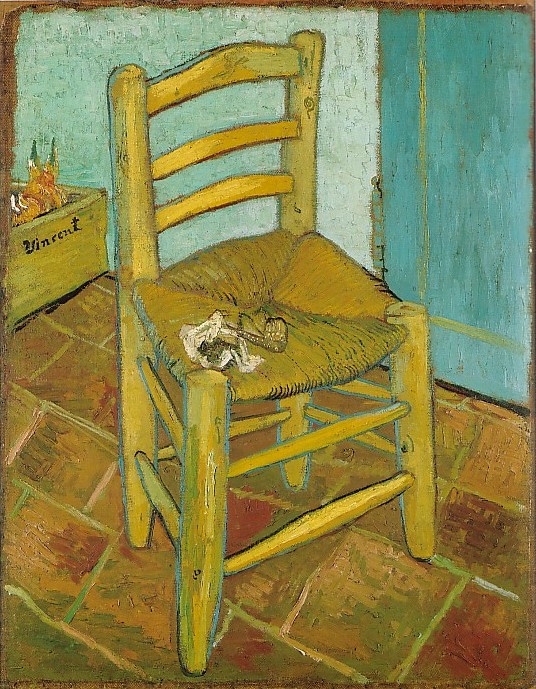  *Feasibility of reducing and breaking up university students' sitting time – Pilot trial and process evaluation* | **Intervention workbook**  **Principal Investigator:**  Oscar Castro Serrano  PhD candidate  Physically Active Lifestyles Research Group (USQ PALs)  Institute for Resilient Regions  University of Southern Queensland  Email: Oscar.CastroSerrano@usq.edu.au  Mobile: +61 (0) 467 030 290 |
| --- | --- |

Introduction --------------------------------------------------------------------------------------------- 3

Activity 1: Normative feedback on sitting behaviour -------------------------------------------- 5

Activity 2: Decisional balance exercise ------------------------------------------------------------- 5

Activity 3: Suggested strategies ---------------------------------------------------------------------- 6

References

Appendix 1. Goal setting

Appendix 2. Pomodoro technique

Appendix 3. Visual cues and apps

Authorship

This workbook has been developed by Oscar Castro in collaboration with his supervisory team. Oscar completed his BSc in psychology at the University of Valencia (Spain), and his MSc in sport and exercise psychology at the University of Jyväskylä (Finland). He is currently a PhD researcher in the area of health behaviour change at the USQ Physically Active Lifestyles Research Group (USQ PALs).


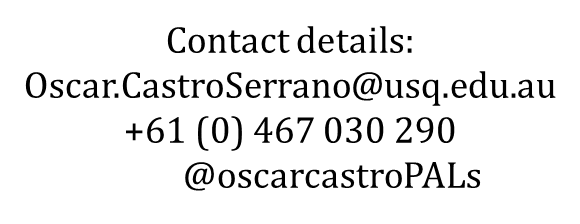


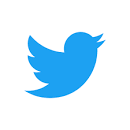


**Introduction**


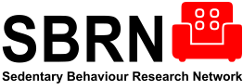
What is sedentary behaviour? Waking activities characterized

by low energy expenditure and undertaken in a sitting or

reclining posture (e.g., reading, watching television, or driving).


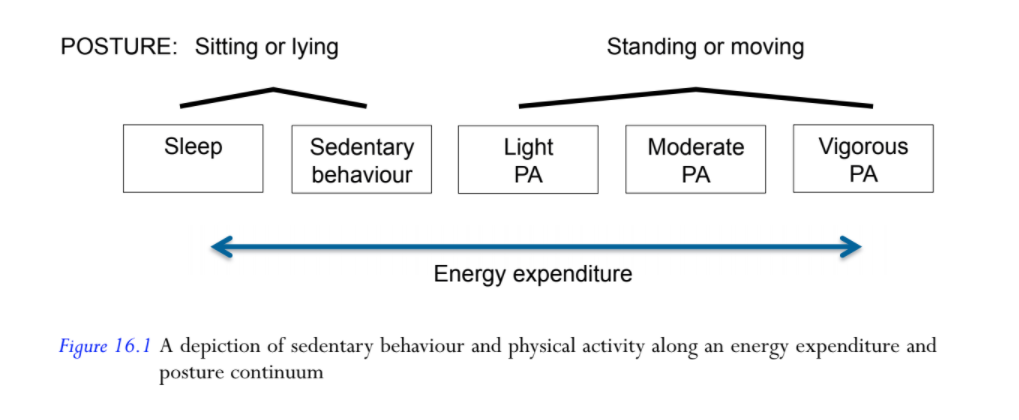
 A depiction of sedentary behaviour and physical activity along an energy expenditure and posture continuum.

**
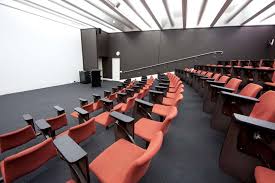

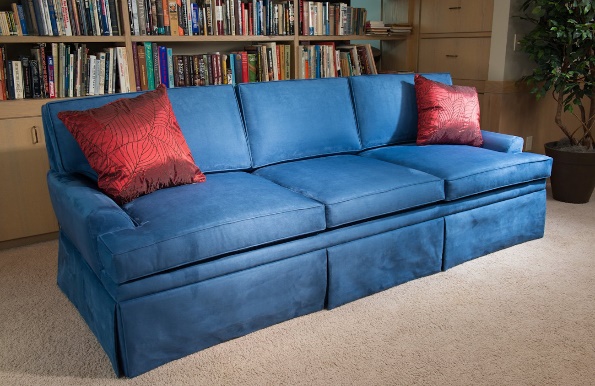
 Occupational sitting time Non-occupational sitting time**

**
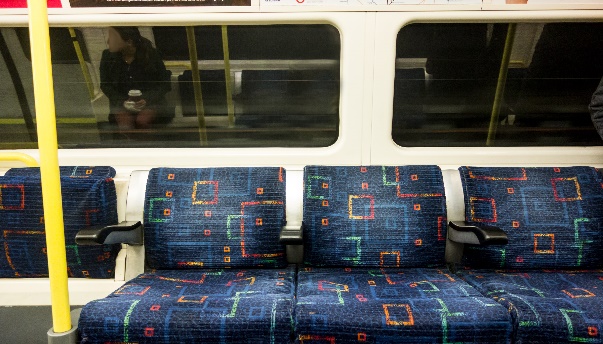
**
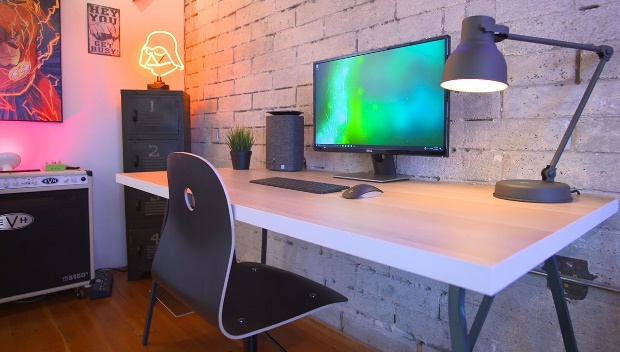


**
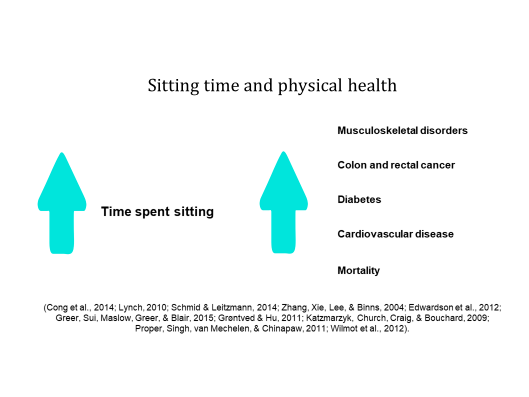
**

**
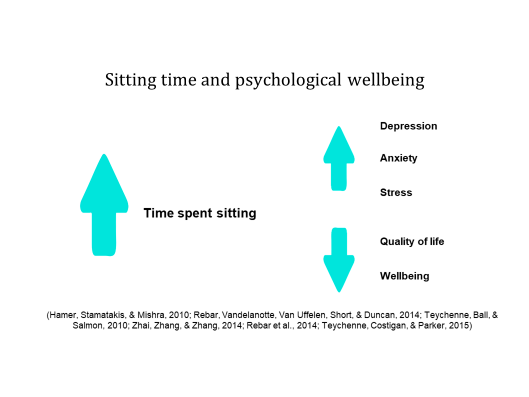
**

**
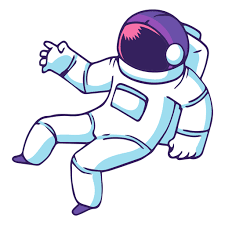

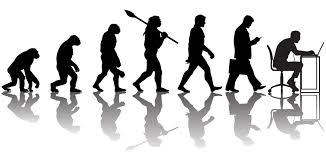
**

**
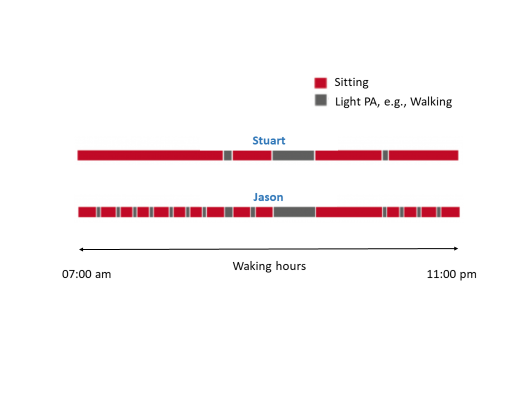

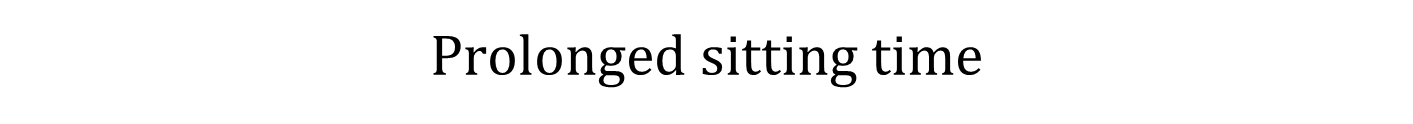
**

**
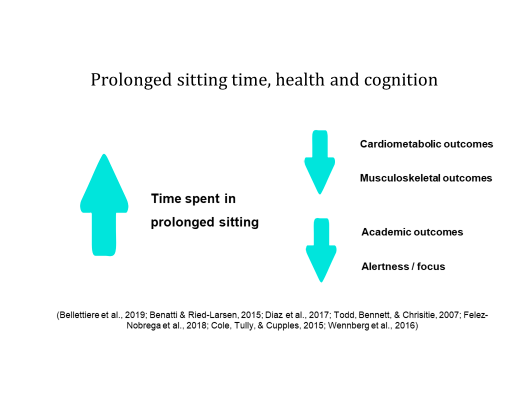
**

**
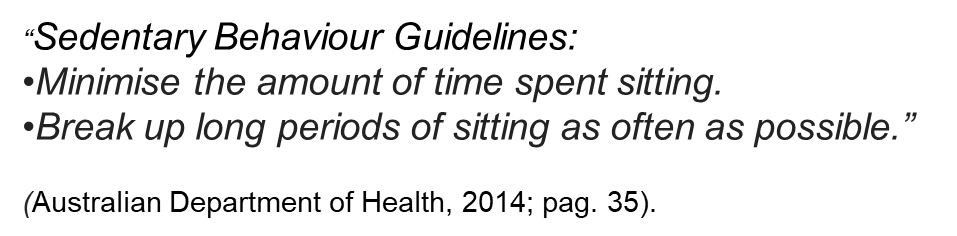
**

**
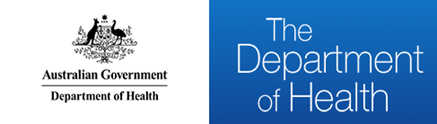
**

**
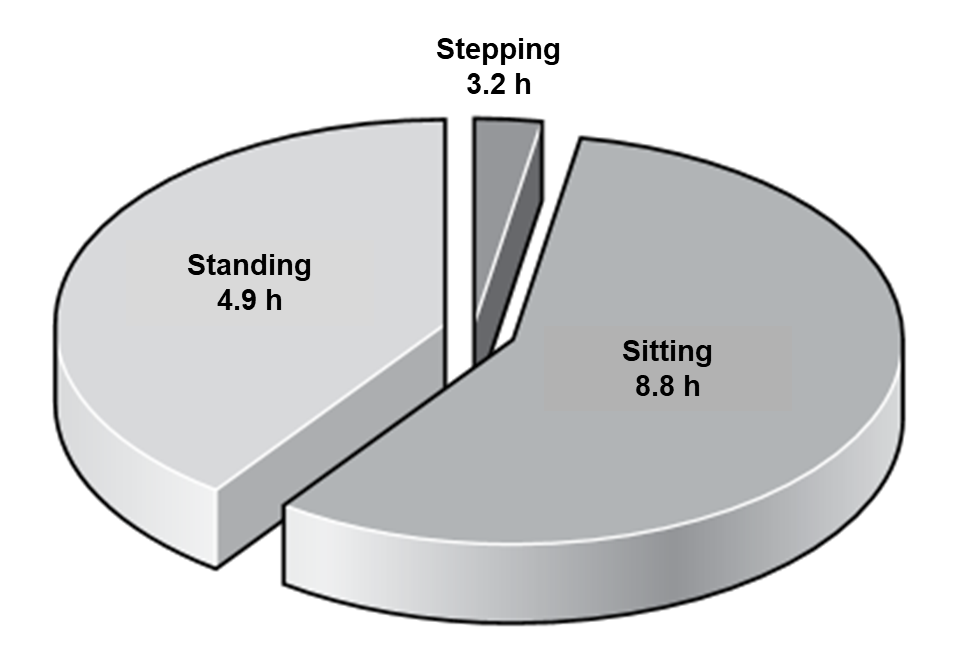

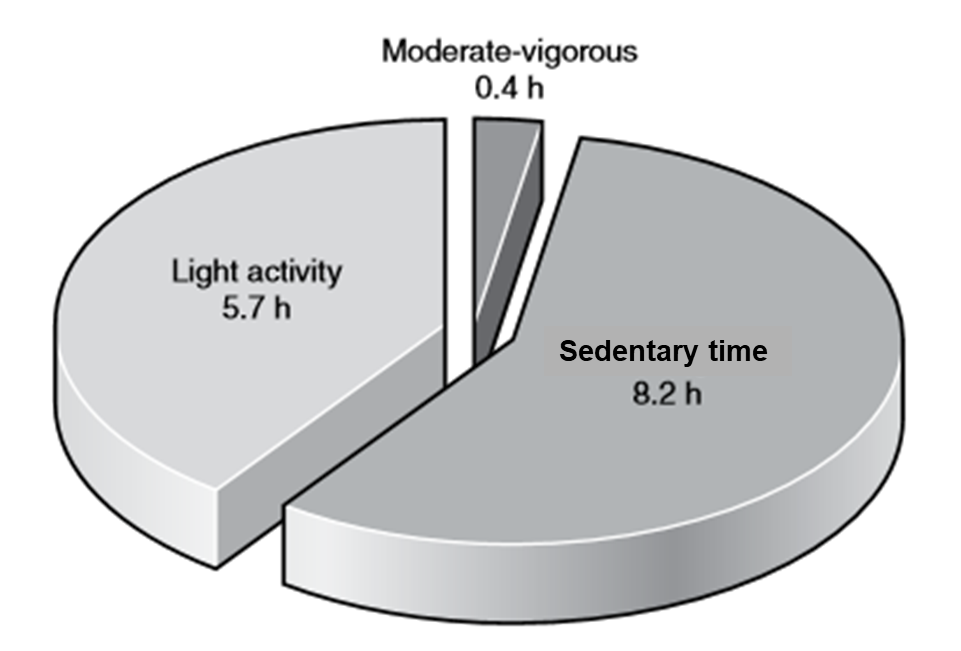
Activity 1: Normative feedback on sitting behaviour**

ActivPAL-derived data from a sample of 741 Australian adults (Healy et al., 2015).

**
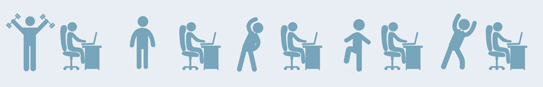
**

**Activity 2: Pros and cons**

You have had the chance to know more about sitting time and your own sitting behaviour over the past week. How do you feel about it? Are you surprised about the amount of time you spent sitting? Is there something you would like to change?

When we think about making changes, most of us don’t really consider all “sides” in a complete way. Instead, we often do what we think we “should” do, avoid doing things we don’t feel like doing, or just feel confused or overwhelmed and give up thinking about it at all.

Thinking through the pros and cons of both changing and not making a change is one way to help us make sure we have fully considered all possible options. This exercise will help you look at the good things and less good things about reducing and breaking up your sitting time.

**
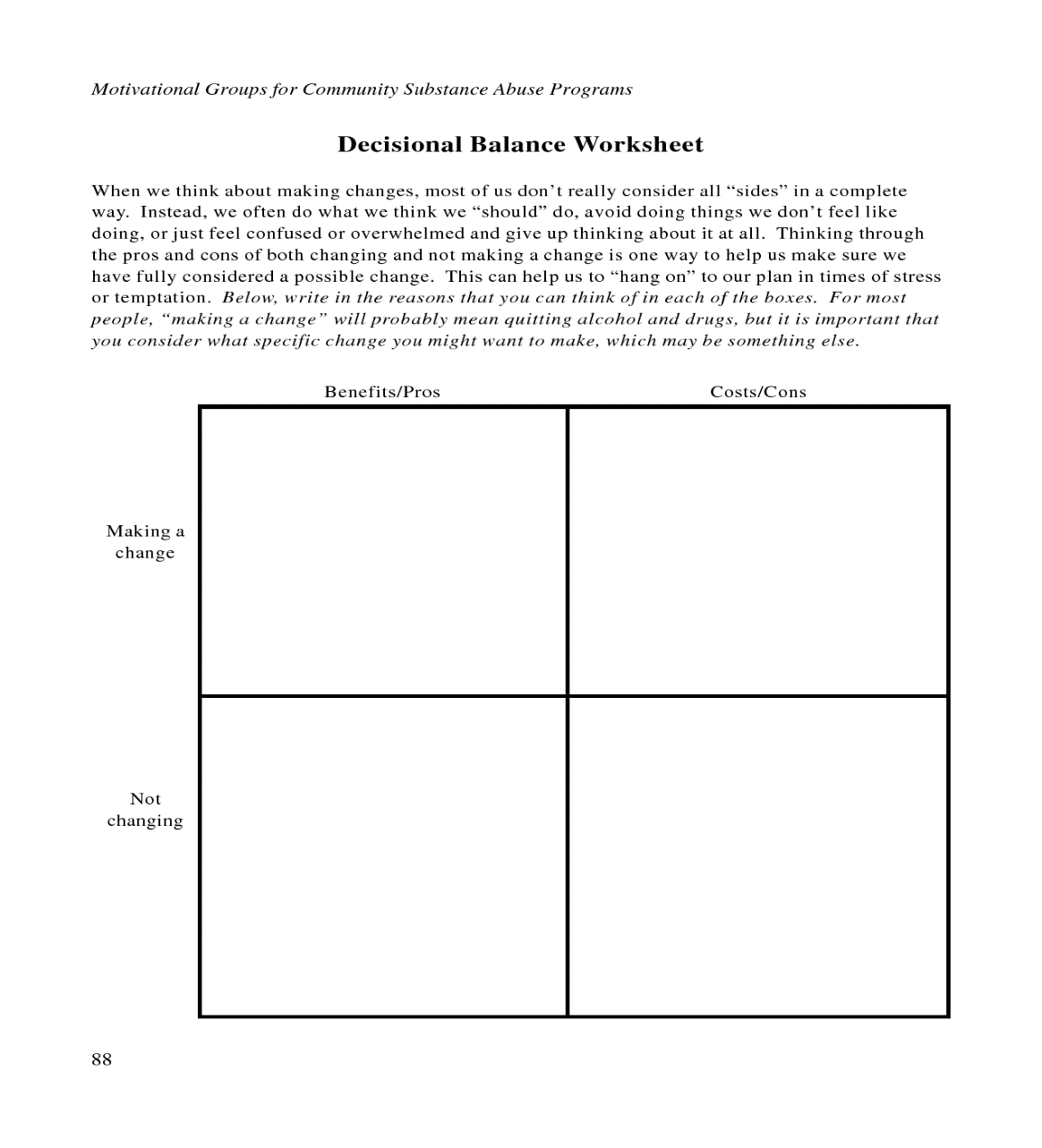
**

**Activity 3: Suggested strategies**

If you consider that reducing and breaking up your sitting time might be a good option for you, here is a list of strategies used by other university students:

- **Ask yourself if you actually need to sit.** Much of our sitting is ‘mindless sitting’ – we don’t actually have to be sitting down, we just do it because it’s the default position. Look at your day, and see what tasks could just as easily be done standing or walking. For example, talking on the phone or waiting in the bus stop. Some university-related activities, like reading or checking your emails in the phone, can also be undertaken standing up!
- **Try new things.** When it comes to breaking up your sitting time, walking around for a few minutes is always a good choice. However, a wide range of activities can serve for the same purpose, get creative! Skipping, jumping jacks, or even dancing.
- **Take the long way.** If you need to change room between lessons, take a longer route to get to your next class. This can apply to toilet breaks as well. And if the new route includes stairs – even better!
- **Schedule reminders.** Your computer or phone can be valuable allies to remind you about standing and moving. If you listen to music while studying, a playset with a set duration can help too. Some popular physical activity trackers provide tactile feedback after a period of sustained inactivity (Fitbit, Garmin). Similarly, you can use environmental signposting in specific contexts to trigger breaks. For example, placing visuals cues such as posters or post-its in your desk will remind you to move more often (see appendices for some example posters).
- **Set goals**. Health behaviour change is challenging, but there are strategies that individuals can use to facilitate their behaviour change efforts. Goal setting is a self-regulation strategy that assists individuals to identify specific behaviours to change and how to go about doing so (for an example of goal setting, see appendices).
- **Make it social.** If you study with others, you can schedule your movement breaks together. That will make them more enjoyable! If you need to do a group assignment with other students, think about stepping away from the library and scheduling some walking meetings. Not only will this give you the opportunity to get some fresh air, but you’ll be able to beat that afternoon slump. Also, if you are in the library and need to talk with other students, walk over to them instead of emailing or texting them.
- **Stay hydrated.** Make regular trips to the kitchen to refill your water glass and add a few steps into your study sessions. Actually, having a coffee or snack break could be a good way to reward yourself and make movement breaks more motivating.
- **Plan in some active time when you are usually sedentary.** In the evenings, consider an exercise class, going for a walk or cooking a healthy meal from scratch. You could even incorporate active time into your commute, parking your car further away or walking part of the journey before you hop on the bus. Home duties and active hobbies such as gardening and DIY are also great options to move more and sit less.
- **Look for opportunities.** Is there any standing desk alternatives available to you? For example, in the library or in some lecture theatres. If so, don’t be shy and give it a try; these alternatives can boost your productivity.
- **Get away from the screens.** Most of our interactions with screen devices involve sitting. Look for ways to reduce screen time in your daily life, for example, limiting your time spent surfing the net and social networking. This will not only potentially reduce your sitting time but will also make you feel more connected with your physical and social environment. Getting up in every ad break when watching TV might also be a useful strategy.
- **Listen to your body.** Stand up when you feel tired or uncomfortable.
- Get started with the **Pomodoro technique** while studying (see appendices).

What do you think about these strategies? Are these applicable to you? Feel free to develop your own specific strategies! Be creative, the point is finding effective ways to reduce and break up your sitting time for a better health (Sit less – move more and more often).

**References**

Australian Department of Health (2014). Australia’s physical activity and sedentary behaviour guidelines for adults (18–64 years). Canberra, Australia.

Bellettiere J., LaMonte M. J., & Evenson K. R. (2019). Sedentary behavior and cardiovascular disease in older women. Circulation 2019; 13: 1036-1046. doi:10.1161/CIRCULATIONAHA.118.035312

Benatti F.B. & Ried-Larsen M. (2015). The effects of breaking up prolonged sitting time: A review of experimental studies. Med Sci Sports Exerc; 47: 2053-2061.

Cole J.A., Tully M.A., & Cupples M. E. (2015). ‘They should stay at their desk until the work’s done’: A qualitative study examining perceptions of sedentary behaviour in a desk-based occupational setting. BMC Res Notes; 8: 105. doi:10.1186/s13104-015-1670-2

Cong, Y., Gan, Y., Sun, H., Deng, J., Cao, S., Xu, X., & Lu, Z. (2014). Association of sedentary behaviour with colon and rectal cancer: A meta-analysis of observational studies. British Journal of Cancer, 110, 817-826.

Diaz K.M., Howard V.J., & Hutto B. (2017). Patterns of sedentary behavior and mortality in US middle-aged and older adults: A national cohort study. Ann Intern Med; 167: 465-475.

Edwardson, C. L., Gorely, T., Davies, M. J., Gray, L. J., Khunti, K., Wilmot, E. G., . . . Biddle, S. J. (2012). Association of sedentary behaviour with metabolic syndrome: A meta-analysis. PloS One, 7, 34916.

Felez-Nobrega M, Hillman CH, Dowd KP et al. ActivPAL™ determined sedentary behaviour, physical activity and academic achievement in college students. J Sports Sci 2018; 36: 2311–2316. doi:10.1080/02640414.2018.1451212

Greer, A. E., Sui, X., Maslow, A. L., Greer, B. K., & Blair, S. N. (2015). The effects of sedentary behavior on metabolic syndrome independent of physical activity and cardiorespiratory fitness. Journal of Physical Activity and Health, 12, 68-73.

Grøntved, A., & Hu, F. B. (2011). Television viewing and risk of type 2 diabetes, cardiovascular disease, and all-cause mortality: A meta-analysis. Jama, 305, 24482455.

Healy, G. N., Winkler, E. A., Owen, N., Anuradha, S., & Dunstan, D. W. (2015). Replacing sitting time with standing or stepping: associations with cardio-metabolic risk biomarkers. European heart journal, 36, 2643-2649.

Hamer, M., Stamatakis, E., & Mishra, G. D. (2010). Television-and screen-based activity and mental well-being in adults. American Journal of Preventive Medicine, 38, 375.

Katzmarzyk, P. T., Church, T. S., Craig, C. L., & Bouchard, C. (2009). Sitting time and mortality from all causes, cardiovascular disease, and cancer. Medicine and Science in Sports and Exercise, 41, 998-1005. doi:10.1249/MSS.0b013e3181930355

Lynch, B. M. (2010). Sedentary behavior and cancer: A systematic review of the literature and proposed biological mechanisms. American Society of Preventive Oncology, 19, 2691-2709. doi:10.1158/10559965.EPI-10-0815

Proper, K. I., Singh, A. S., Van Mechelen, W., & Chinapaw, M. J. (2011). Sedentary behaviors and health outcomes among adults: A systematic review of prospective studies. American Journal of Preventive Medicine, 40, 174-182.

Rebar, A. L., Vandelanotte, C., Van Uffelen, J., Short, C., & Duncan, M. J. (2014). Associations of overall sitting time and sitting time in different contexts with depression, anxiety, and stress symptoms. Mental Health and Physical Activity, 7, 105-110.

Teychenne, M., Ball, K., & Salmon, J. (2010). Sedentary behavior and depression among adults: A review. International Journal of Behavioral Medicine, 17, 246-254.

Teychenne, M., Costigan, S. A., & Parker, K. (2015). The association between sedentary behaviour and risk of anxiety: A systematic review. BMC Public Health, 15, 513.

Todd A.I., Bennett A.I., & Chrisitie C. J. (2007). Physical implications of prolonged sitting in a confined posture: A literature review. J Ergo S Afr; 19: 7–21.

Tremblay M. S. (2017). Sedentary Behavior Research Network (SBRN) – Terminology Consensus Project process and outcome. Int J Behav Nutr Phys; 14: 75.

Schmid, D., & Leitzmann, M. F. (2014). Television viewing and time spent sedentary in relation to cancer risk: A meta-analysis. Journal of the National Cancer Institute, 106. doi:10.1093/jnci/dju098

Wennberg P., Boraxbekk C. J., Wheeler M. (2016). Acute effects of breaking up prolonged sitting on fatigue and cognition: A pilot study. BMJ open; 6: e009630.

Wilmot, E. G., Edwardson, C. L., Achana, F. A., Davies, M. J., Gorely, T., Gray, L. J., . . . Biddle, S. J. (2012). Sedentary time in adults and the association with diabetes, cardiovascular disease and death: Systematic review and meta-analysis. Ann Intern Med, 162, 123-132. doi:10.7326/M14-1651

Zhai, L., Zhang, Y., & Zhang, D. (2015). Sedentary behaviour and the risk of depression: A meta-analysis. British Journal of Sports Medicine, 49, 705-709. doi:10.1136/bjsports-2014-093613

Zhang, M., Xie, X., Lee, A. H., & Binns, C. W. (2004). Sedentary behaviours and epithelial ovarian cancer risk. Cancer Causes and Control, 15, 83-89.

**Appendix 1. Goal setting**

In order to reduce and/or break up my sitting time in the following week (including occupation and non-occupational sitting), I will try to:

1. *Reduce my sitting time during leisure activities by 1 hour each day.*

When will you do it?

1. *In the evening, right after coming back home from the uni.*

How will you do it?

1. *I’ll park the car further away from home and walk a bit longer. I’ll also stand up when*

*texting to my friends and checking social media on the mobile phone.*

**Remember to set SMART goals! Specific, Measurable, Attainable, Relevant, Timely.**

**Appendix 2. Pomodoro technique**


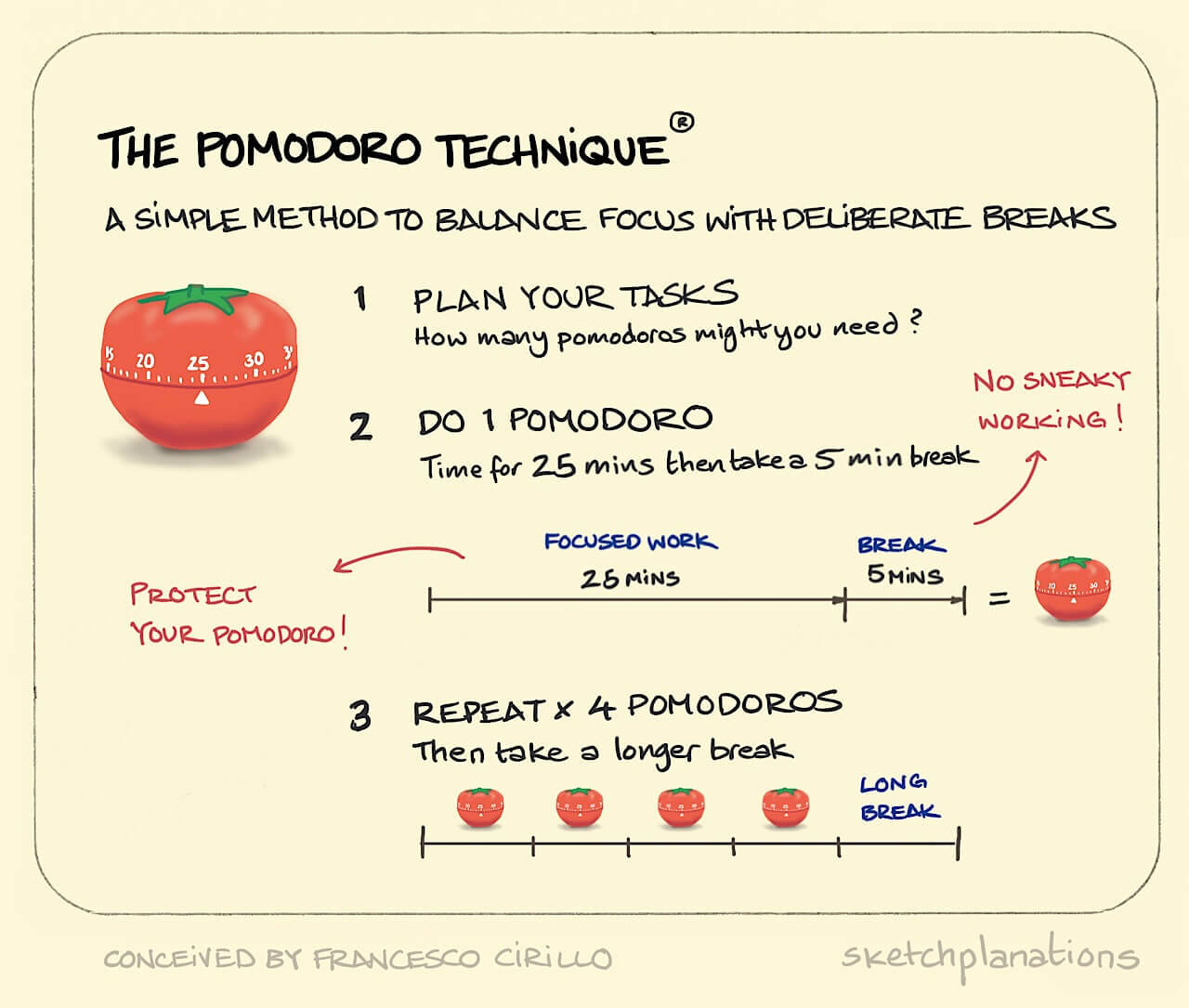


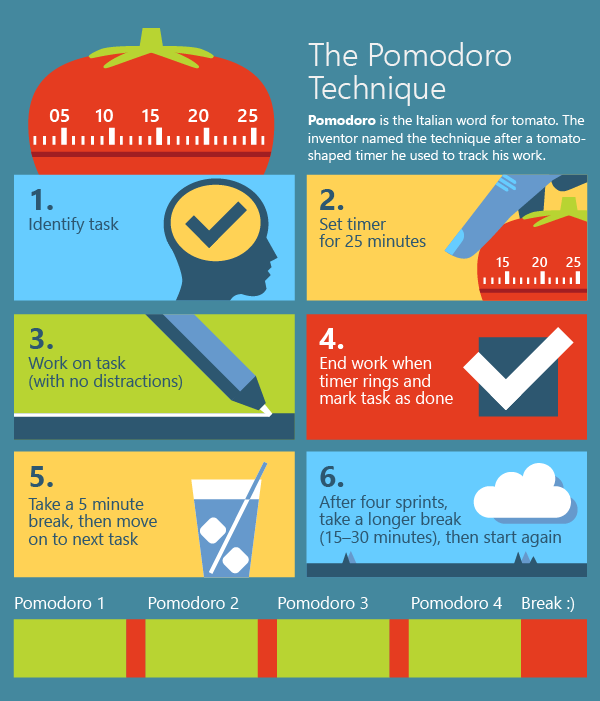


**Appendix 3. Visual cues and apps**

You can find a variety of posters related to sitting behaviour, as well as other useful resources, in the following webpage:

<https://www.movemoresitless.org.au/download-posters/>

Moreover, many **free** apps can help you reducing sitting and introducing physical activity into your daily life.

Mobile device reminder apps (iPhones and iPads)

**Move - Daily Activity to Stay Healthy**: Get reminded every so often to stand up and do a tiny exercise. Over 300 random workouts keep it exciting (more added every day). Create a healthy habit through occasional reminders. Pick when you do workouts and how often. Choose your reminder sound. Create your own exercises to be absolutely anything you want.

**Stand Up! The Work Break Timer**: Completely customizable to your work schedule. Set it and forget it. Set your reminder interval to any five-minute increment between five minutes and two hours. The header shows you at a glance how you're doing, and how long to your next alarm. Limit alarms to your office location so it doesn't bug you when you go out to lunch.

Mobile device reminder apps (Android phones and tablets)

**Randomly RemindMe**: Do you have trouble drinking enough water during the day? Maybe you want a reminder to step away from the computer for a bit. How about a reminder to stop and do push-ups? Trying to make a habit? Or break one? This app will let you set any number of custom reminders that will remind you throughout the day for that and a whole lot more.

**Hourly fitness**: A simple, time-friendly app, aimed at those who want to be more active throughout the day. Hourly Exercise will remind you to get up and do a basic exercise, once every hour; You may do it, or skip it if you can't do it right then and there.

Computer reminder apps (Windows)

**Awareness**: This is an app that sets a timer in your menu bar, counting the time you’ve been active in your computer. Once you reach a specific period of time without taking a break, the sound of a bowl will play, reminding you that it’s time to step away for a while. If the app doesn’t detect activity in your computer after a set period of time, the timer will reset; however, if you keep working without taking a break, the next time the timer goes off, the sound of the bowl will be played twice, and so forth. The app even includes a link to a website where you can find some useful ideas on how to take advantage of your breaks.

**Big Stretch Reminder**: Big Stretch is a simple reminder tool that prompts you to take regular breaks and helps prevent the symptoms that come from sitting too long. Alternatively it can be a simple reminder program to tell you when it’s your coffee break!

**Workrave**: This program reminds you to take microbreaks throughout the day and can also help you limit your computer usage while at home. It has settings that let you configure it in a way that works best for you including when to take breaks and how long they should be. This program also gives you examples of exercises that you can do while on a break.

Computer reminder apps (Mac)

**Awareness**: Awareness helps you become more aware of time spent on the computer by playing the sound of a Tibetan singing bowl to mark every hour of continuous computer use. It also displays how long you’ve been using your computer without a break in the menu bar. Awareness will never nag you or force you to stop using the computer. Just take a five-minute break whenever you are ready, and Awareness will sense it.

**Time Out**: Time Out has two kinds of breaks: a "Normal" break and a "Micro" break. You can disable either kind of break if desired, and the breaks are automatically paused when you go away from your computer, and can be reset when you come back. You can configure how long each kind of break lasts, and how long between breaks. Each Time Out is announced via the screen slowly dimming, with related graphics materializing, and when the break is complete, it fades out again.
