# Supplementary material for: Feasibility of Reducing and Breaking Up University Students' Sedentary Behaviour: Pilot Trial and Process Evaluation
Source: Front Psychol. 2021 Jun 10;12:661994. doi: 10.3389/fpsyg.2021.661994 (PMC8222591; doi:10.3389/fpsyg.2021.661994)
Supplement: Supplementary file 2 [file Table_2.DOCX]

**Supplementary file 2**: Data collection and intervention text messages

Data collection messages

- Hi! This is just a reminder for you to attach the ActivPAL this morning. Please follow the instructions provided in your activity monitor pack. If you have any doubts or concerns, please contact Oscar on +61 (0) XXX XXX XXX or oscar.castroserrano@usq.edu.au. Thank you for joining the study!
- Hi! This is just a reminder for you to fill in the ActivPAL Log and the Sedentary Time Questionnaire today. Try to do it as close to your sleeping time as possible, or at 12AM midnight (whichever comes first). Thank you for your participation!
- Hi! It’s been 6 days already! Time flies, isn’t it? You can remove your ActivPAL device tomorrow, once you wake up. Please remember to return both the device and the daily logs to our research team during the scheduled appointment. In case you have to reschedule the appointment please contact Oscar on +61 (0) XXX XXX XXX or oscar.castroserrano@usq.edu.au
- Hi! This is just a friendly reminder for tomorrow’s appointment with Oscar (ActivPAL study). Campus: XXX Time: XXX Duration: XXX. In case you have to reschedule the appointment or there are some last-minute changes please contact Oscar on +61 (0) XXX XXX XXX or oscar.castroserrano@usq.edu.au. Thank you for your participation!

Intervention messages

*Break up messages (nudges)*

- If you've been sitting for more than an hour consider getting up and move! Try walking around or doing some light stretching.
- Warrior, tree, frog, cobra, triangle, cat. Do you know what these words have in common? All of them are yoga poses. You can give them a try! Get creative when it comes to breaking up your sitting time.
- Do you fancy a drink or a snack? Go grab them! This can be a great opportunity to reward yourself and make movement breaks more motivating.
- Have you gone for a walk yet today? It's not too late if you haven't! there are many opportunities to move more throughout the day.
- How about breaking up your sitting now with a 3-minute break? Squats, lunges and jumping jacks are all great ways to kill 3 mins! Try a minute of each and see how many you can do.
- Thirsty? Make regular trips to the kitchen to refill your water glass and add a few steps into your study sessions.

*Health-related messages*

- Effects of sitting aren’t just long term. As soon as you sit, the electrical activity in the leg muscles shuts off and the enzymes that help break down fat drop. Find ways to move more and sit less.
- Walking burns 5 times the calories that sitting does. Take every opportunity to walk around!
- People with sitting jobs have twice the rate of cardiovascular disease as people with standing jobs. Sit less – move more and more often for a better health.
- University students are a ‘high-sitting’ group within the general population, so it’s important that you pay special attention to your sitting patterns.
- As Bob Marley says, "Get up, Stand up, Stand up for your health". Okay those aren't quite the lyrics, but you get it. Sit less and move more for a better health!
- Humans are built to stand upright. Sitting for long periods can lead to pain and stiffness in your back, neck and shoulders.

*Psychological wellbeing and productivity messages*

- Interrupting prolonged sitting is important for mental performance. Many university students report breaking up their sitting to ‘refresh’ their mind and enhance productivity.
- In a recent study examining the relationship between accelerometer-based sedentary behaviour and academic achievement, it was found that university students who interrupted their sitting time every 30 mins had higher academic scores. Why not give it a try?
- While attention typically decays after 30 minutes performing a task, short active breaks may temporarily restore attention levels. Use your breaks to perform at an optimal level for longer!
- Breaking up sitting time with short walking breaks has been shown to counteract mental fatigue, in comparison with continuous sitting.
- Introducing more movement into your daily life will trigger a feel-good response. And remember, something is better than nothing and more is better than less.
- Sitting less and moving more is a good opportunity to spend more time outdoors. Being outside can lead to significant psychological benefits, such as reduced stress.

*Strategies to reduce and break up sitting*

- Much of our sitting is automatic or ‘mindless sitting’ – we don’t actually have to be sitting down! We just do it because it’s the default position. Look at your day and see what activities you could do standing or walking, rather than sitting.
- Need a tip to sit less? Try walking to uni or the store if you live close enough, or if you drive, park further away from where you are going. It'll add some steps to your day and give you some nice fresh air!
- Sometimes it might be difficult to remember taking breaks. University activities are very absorbing! Your computer or phone can be valuable allies to remind you about standing and moving.
- You would like to move more and sit less but don’t know how to start? Set a specific, easily achievable goal for today and try to scale it up progressively. Thinking small is the secret to big success.
- Try to engage other people in your ‘move more sit less’ endeavours. This will make them more pleasant. Also, remember that your own behaviour may be an example for other students to reduce and break up their sitting time.
- You can use habit formation strategies to change your sitting patters. For example, try to consistently pair standing breaks with daily habits such as texting on the phone or drinking coffee.
