# Supplementary material for: Feasibility of Reducing and Breaking Up University Students' Sedentary Behaviour: Pilot Trial and Process Evaluation
Source: Front Psychol. 2021 Jun 10;12:661994. doi: 10.3389/fpsyg.2021.661994 (PMC8222591; doi:10.3389/fpsyg.2021.661994)
Supplement: Supplementary file 3 [file Table_3.DOCX]

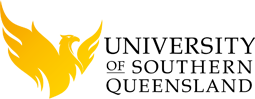


**Return Appointment: _____ am / pm on**

**_____/_____/_____**

**Activity Monitor Instructions**

**& Daily Log**

**Please keep this booklet in a safe place so you can return it to us**

ActivPAL serial # __ __ __ __ __ __ __

Participant ID: __ __ __ __ __ __ __

**If you have any questions or concerns,**

**please contact Oscar on +61 (0) XXX XXX XXX or oscar.castroserrano@usq.edu.au**

**Attaching the monitor**

1. Sit down on a chair when attaching the monitor so that your thigh is in a horizontal position. This will also make it easier to find the top of your thigh (the crease between your leg and your upper body).
2.
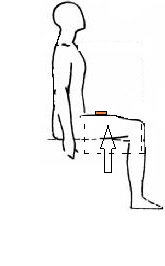
The monitor is to be attached one third of the way down between the top of your thigh and top of your knee. Position the monitor in the midline of your right thigh as shown in the picture.
3. Swab the area where the monitor is to be attached with the provided alcohol pad and let the area dry for a few seconds.
4. Place the monitor in the correct position on the thigh, ensuring that the man on the front of the monitor is standing up (head facing upwards) when you stand up.
5.
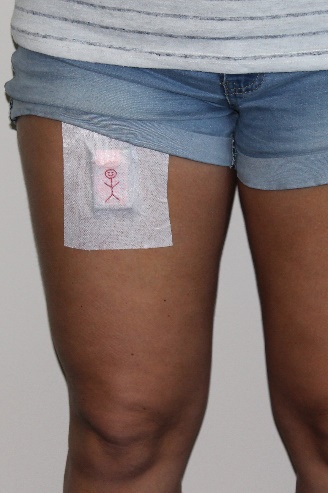
Peel the backing off an adhesive patch (provided in your activity monitor pack) and place it over the monitor. Press the patch onto your skin, starting from the middle out towards the edges and smooth out the air bubbles and wrinkles as much as possible to ensure that the monitor is firmly secured to your thigh.
6. Please wear the monitor continuously (24 hours/day) for 6 consecutive days, removing it on the morning of day 7. The thigh monitor is water resistant (to 1m) so you can wear it whilst showering and swimming in a pool, but please do not wear it in the ocean in case it falls off. The activity monitor can be worn through airport security.

**If you need to change the adhesive patch**

During your wear time, you may need to change the adhesive patch which attaches the monitor to your thigh. To do this:

- - Remove the monitor from your thigh (note that this may cause some slight discomfort) and peel the adhesive patch off the monitor.
  - With an alcohol prep pad provided in your Monitor Pack, thoroughly wipe down the monitor and the area of your leg where the monitor was attached and follow the same procedure as explained.

**Other notes**: Although it is unlikely, skin irritations due to the adhesive tape may occur. If this happens, attach the monitor to the other (left) leg. If you still continue to experience irritation contact the research team for further instructions. The Thigh Monitor will emit a green flash every 6 seconds. This is an indication that it is working and recording data. If you need more info on how to fit the ActivPAL, try this Youtube tutorial:

Video: *activPAL activity monitor* - <https://www.youtube.com/watch?v=CHCCX2GW3DM>

**What else do I need to do?**

It is very important that you fill in the **ActivPAL Log** and the **Sedentary Time Questionnaire** every day for the next 6 days while you are wearing the monitor.

**How to fill in the daily ActivPAL log**

- The log is divided into 6 days. Please complete each question for all of the 6 days. Please try and be as accurate as possible—record the exact times if you can, or at least to the nearest 10 minutes of your estimated times.
- Please fill in each daily log at the end of the day, as close to your sleeping time as possible, or at 12AM midnight (whichever comes first).
- Record the time that you **woke up** and the time that you actually **got out of bed** (these times may be the same for most people). We ask for these two times because people sometimes spend time in bed before going to sleep or getting up and we are interested in distinguishing between actual sleeping time and time in bed before sleep or once awake, for example going to bed and watching TV or reading for an hour before going to sleep.
- Please also record your **‘occupational’ time**. That is, the time you spend doing any university-related activity (e.g., lectures, tutorials, meetings, independent study, completing assignments, reading, sending emails, etc), unless this was a very short time (less than 15 minutes). Do not include information about any other occupation you may have (e.g., a part-time job).
- If you **remove the device** for longer than 15 minutes during the day, please note down the time that you removed the device, the time that you re-attached it and the reason why you removed the device. This is particularly important, as we cannot tell from the data whether you are lying down or you have just removed the device (the data looks the same when we look at it).

- Then estimate at what time you will **get into bed** and the time that you actually will **go to sleep** time (these times may be the same for most people). This is important as the monitor cannot tell the difference between asleep and awake times, and we are only interested in your activity while you are awake.
- There is also a space for you to make **comments**. It is useful for us to know if you have had any skin irritations, accidentally worn the monitor upside down or any other information that you think we should know. Once you have completed your 6 days of wear, please return both the device and the daily log to our research team during the scheduled appointment.

**How to fill in the daily Sedentary Time Questionnaire?**

- Please complete the questionnaire for all of the next 6 days. Please try and be as accurate as possible—record the exact amount of time if you can, or at least the closest amount you can think of.
- Please fill in each daily log at the end of the day, as close to your sleeping time as possible, or at 12AM midnight (whichever comes first).
- You will be asked about when you may have been sitting or lying down in the following domains listed below. For each of these, only count the time this was your main activity. Refer to the following instructions on how to properly account for sitting/lying time each day.
- **Studying**: time spent doing any university-related activity. Examples: lectures, tutorials, meetings, independent study, completing assignments, reading, sending emails, etc.
- **Work**: paid position only. Examples: babysitting, sitting at the reception, minding a stall/shop, data entry/administrative paper work, tutoring, etc.
- **Transport**: travelling from one place to another. Please include sitting and waiting for transport. Do not include any time you were standing up while travelling or waiting.
- **Television Viewing**: watching TV or DVDs or playing games on the TV, such as PlayStation/Xbox. This includes if you watch TV in bed. Do not include watching TV on your computer, such as YouTube.
- **Computer, Internet, Electronic Games**: include time spent playing games on your phone/tablet, using the internet or activities that were not for studying or working purposes, like Facebook, Twitter, Skype, YouTube, online-shopping, etc.
- **Sitting for Leisurely Reading**: include recreational reading, but do not include time spent reading for paid work or for study.
- **Sitting for Eating**: include eating and drinking, meals and snack breaks. If you went out to eat with friends, consider this sitting for socializing and not sitting for eating.
- **Sitting for Socializing**: include time with friends and family. Include time on the telephone. Do not overlap with other domains such as eating. If you went out to eat with friends, considering this socializing time and not eating time.
- **Sitting/Lying for Other Purposes**: any sitting/lying time that has not been accounted for in the previously listed domains. This may include hobbies, listening to music, playing an instrument or sitting for religious purposes.
- Do not complete domains as they happen throughout the day, as this may cause you to miss activities that occur later in the day. Please note that this does not include sleep, either in bed or if you fell asleep while doing another activity, for example watching TV.

| **DAY 1:** | **ActivPAL log** | Date: _____/ _____ / _______ |
| --- | --- | --- |
| **Sleep** | What time did you wake up today? _____:____ am / pm  What time did you get out of bed? _____:____ am / pm | |
|  | What time will you get into bed? _____:____ am / pm  What time will you go to sleep today? _____:____ am / pm | |
| **University** | Did you carry out any university-related activity today (e.g., lectures, tutorials, meetings, independent study, completing assignments, reading, sending emails, etc)? Please record all the times you spent doing these activities: | **□ No □ Yes** |
|  | **Time Started Time Finished** | |
|  | : am / pm : am / pm  : am / pm : am / pm  : am / pm : am / pm  : am / pm : am / pm  : am / pm : am / pm  : am / pm : am / pm  : am / pm : am / pm | |
| **Monitor** | Did you remove your thigh monitor today for more than 15 minutes? | |
|  | **□ No □ Yes** *If yes, please note time off/on:*  Time off: ____:____ am / pm Time on: ____:____ am / pm  Time off: ____:____ am / pm Time on: ____:____ am / pm | |
| **Other comments:** |  | |

| **DAY 1:** | **Sedentary Time Questionnaire** | Date: _____/ _____ /______ |
| --- | --- | --- |
| **Sitting for study** | How long were you sitting or lying down while studying today?   \|  \|  \| hours \|  \|  \| minutes \| \| --- \| --- \| --- \| --- \| --- \| --- \| | |
| **Sitting for work** | How long were you sitting or lying down at your workplace or working from home today?   \|  \|  \| hours \|  \|  \| minutes \| \| --- \| --- \| --- \| --- \| --- \| --- \| | |
| **Sitting for Transport** | How long were you sitting or lying down for transport today?   \|  \|  \| hours \|  \|  \| minutes \| \| --- \| --- \| --- \| --- \| --- \| --- \| | |
| **Television Viewing** | How long were you sitting or lying down to watch TV today?   \|  \|  \| hours \|  \|  \| minutes \| \| --- \| --- \| --- \| --- \| --- \| --- \| | |
| **Computer, Internet, Electronic Games** | How long were you sitting or lying down while using the computer today?   \|  \|  \| hours \|  \|  \| minutes \| \| --- \| --- \| --- \| --- \| --- \| --- \| | |
| **Sitting for leisurely reading** | How long were you sitting or lying down while reading during your leisure time today?   \|  \|  \| hours \|  \|  \| minutes \| \| --- \| --- \| --- \| --- \| --- \| --- \| | |
| **Sitting for eating** | How long were you sitting or lying down while eating and drinking today?   \|  \|  \| hours \|  \|  \| minutes \| \| --- \| --- \| --- \| --- \| --- \| --- \| | |
| **Sitting for socializing** | How long were you sitting or lying down to socialize with family and friends today?   \|  \|  \| hours \|  \|  \| minutes \| \| --- \| --- \| --- \| --- \| --- \| --- \| | |
| **Sitting for other purposes** | How long were you sitting or lying down today in other pursuits NOT including the time that you have already logged above?   \|  \|  \| hours \|  \|  \| minutes \| \| --- \| --- \| --- \| --- \| --- \| --- \| | |

Please take a moment to add up your total sitting time for today. Considering how many hours you were awake, does this amount of sitting make sense? If not, please make changes to reflect your true sitting time.

**Thank you for your time**

| **DAY 2:** | **ActivPAL log** | Date: _____/ _____ / _______ |
| --- | --- | --- |
| **Sleep** | What time did you wake up today? _____:____ am / pm  What time did you get out of bed? _____:____ am / pm | |
|  | What time will you get into bed? _____:____ am / pm  What time will you go to sleep today? _____:____ am / pm | |
| **University** | Did you carry out any university-related activity today (e.g., lectures, tutorials, meetings, independent study, completing assignments, reading, sending emails, etc)? Please record all the times you spent doing these activities: | **□ No □ Yes** |
|  | **Time Started Time Finished** | |
|  | : am / pm : am / pm  : am / pm : am / pm  : am / pm : am / pm  : am / pm : am / pm  : am / pm : am / pm  : am / pm : am / pm  : am / pm : am / pm | |
| **Monitor** | Did you remove your thigh monitor today for more than 15 minutes? | |
|  | **□ No □ Yes** *If yes, please note time off/on:*  Time off: ____:____ am / pm Time on: ____:____ am / pm  Time off: ____:____ am / pm Time on: ____:____ am / pm | |
| **Other comments:** |  | |

| **DAY 2:** | **Sedentary Time Questionnaire** | Date: _____/ _____ / _____ |
| --- | --- | --- |
| **Sitting for study** | How long were you sitting or lying down while studying today?   \|  \|  \| hours \|  \|  \| minutes \| \| --- \| --- \| --- \| --- \| --- \| --- \| | |
| **Sitting for work** | How long were you sitting or lying down at your workplace or working from home today?   \|  \|  \| hours \|  \|  \| minutes \| \| --- \| --- \| --- \| --- \| --- \| --- \| | |
| **Sitting for Transport** | How long were you sitting or lying down for transport today?   \|  \|  \| hours \|  \|  \| minutes \| \| --- \| --- \| --- \| --- \| --- \| --- \| | |
| **Television Viewing** | How long were you sitting or lying down to watch TV today?   \|  \|  \| hours \|  \|  \| minutes \| \| --- \| --- \| --- \| --- \| --- \| --- \| | |
| **Computer, Internet, Electronic Games** | How long were you sitting or lying down while using the computer today?   \|  \|  \| hours \|  \|  \| minutes \| \| --- \| --- \| --- \| --- \| --- \| --- \| | |
| **Sitting for leisurely reading** | How long were you sitting or lying down while reading during your leisure time today?   \|  \|  \| hours \|  \|  \| minutes \| \| --- \| --- \| --- \| --- \| --- \| --- \| | |
| **Sitting for eating** | How long were you sitting or lying down while eating and drinking today?   \|  \|  \| hours \|  \|  \| minutes \| \| --- \| --- \| --- \| --- \| --- \| --- \| | |
| **Sitting for socializing** | How long were you sitting or lying down to socialize with family and friends today?   \|  \|  \| hours \|  \|  \| minutes \| \| --- \| --- \| --- \| --- \| --- \| --- \| | |
| **Sitting for other purposes** | How long were you sitting or lying down today in other pursuits NOT including the time that you have already logged above?   \|  \|  \| hours \|  \|  \| minutes \| \| --- \| --- \| --- \| --- \| --- \| --- \| | |

Please take a moment to add up your total sitting time for today. Considering how many hours you were awake, does this amount of sitting make sense? If not, please make changes to reflect your true sitting time.

**Thank you for your time**

| **DAY 3:** | **ActivPAL log** | Date: _____/ _____ / _______ |
| --- | --- | --- |
| **Sleep** | What time did you wake up today? _____:____ am / pm  What time did you get out of bed? _____:____ am / pm | |
|  | What time will you get into bed? _____:____ am / pm  What time will you go to sleep today? _____:____ am / pm | |
| **University** | Did you carry out any university-related activity today (e.g., lectures, tutorials, meetings, independent study, completing assignments, reading, sending emails, etc)? Please record all the times you spent doing these activities: | **□ No □ Yes** |
|  | **Time Started Time Finished** | |
|  | : am / pm : am / pm  : am / pm : am / pm  : am / pm : am / pm  : am / pm : am / pm  : am / pm : am / pm  : am / pm : am / pm  : am / pm : am / pm | |
| **Monitor** | Did you remove your thigh monitor today for more than 15 minutes? | |
|  | **□ No □ Yes** *If yes, please note time off/on:*  Time off: ____:____ am / pm Time on: ____:____ am / pm  Time off: ____:____ am / pm Time on: ____:____ am / pm | |
| **Other comments:** |  | |

| **DAY 3:** | **Sedentary Time Questionnaire** | Date: _____/ _____ / _____ |
| --- | --- | --- |
| **Sitting for study** | How long were you sitting or lying down while studying today?   \|  \|  \| hours \|  \|  \| minutes \| \| --- \| --- \| --- \| --- \| --- \| --- \| | |
| **Sitting for work** | How long were you sitting or lying down at your workplace or working from home today?   \|  \|  \| hours \|  \|  \| minutes \| \| --- \| --- \| --- \| --- \| --- \| --- \| | |
| **Sitting for Transport** | How long were you sitting or lying down for transport today?   \|  \|  \| hours \|  \|  \| minutes \| \| --- \| --- \| --- \| --- \| --- \| --- \| | |
| **Television Viewing** | How long were you sitting or lying down to watch TV today?   \|  \|  \| hours \|  \|  \| minutes \| \| --- \| --- \| --- \| --- \| --- \| --- \| | |
| **Computer, Internet, Electronic Games** | How long were you sitting or lying down while using the computer today?   \|  \|  \| hours \|  \|  \| minutes \| \| --- \| --- \| --- \| --- \| --- \| --- \| | |
| **Sitting for leisurely reading** | How long were you sitting or lying down while reading during your leisure time today?   \|  \|  \| hours \|  \|  \| minutes \| \| --- \| --- \| --- \| --- \| --- \| --- \| | |
| **Sitting for eating** | How long were you sitting or lying down while eating and drinking today?   \|  \|  \| hours \|  \|  \| minutes \| \| --- \| --- \| --- \| --- \| --- \| --- \| | |
| **Sitting for socializing** | How long were you sitting or lying down to socialize with family and friends today?   \|  \|  \| hours \|  \|  \| minutes \| \| --- \| --- \| --- \| --- \| --- \| --- \| | |
| **Sitting for other purposes** | How long were you sitting or lying down today in other pursuits NOT including the time that you have already logged above?   \|  \|  \| hours \|  \|  \| minutes \| \| --- \| --- \| --- \| --- \| --- \| --- \| | |

Please take a moment to add up your total sitting time for today. Considering how many hours you were awake, does this amount of sitting make sense? If not, please make changes to reflect your true sitting time.

**Thank you for your time**

| **DAY 4:** | **ActivPAL log** | Date: _____/ _____ / _______ |
| --- | --- | --- |
| **Sleep** | What time did you wake up today? _____:____ am / pm  What time did you get out of bed? _____:____ am / pm | |
|  | What time will you get into bed? _____:____ am / pm  What time will you go to sleep today? _____:____ am / pm | |
| **University** | Did you carry out any university-related activity today (e.g., lectures, tutorials, meetings, independent study, completing assignments, reading, sending emails, etc)? Please record all the times you spent doing these activities: | **□ No □ Yes** |
|  | **Time Started Time Finished** | |
|  | : am / pm : am / pm  : am / pm : am / pm  : am / pm : am / pm  : am / pm : am / pm  : am / pm : am / pm  : am / pm : am / pm  : am / pm : am / pm | |
| **Monitor** | Did you remove your thigh monitor today for more than 15 minutes? | |
|  | **□ No □ Yes** *If yes, please note time off/on:*  Time off: ____:____ am / pm Time on: ____:____ am / pm  Time off: ____:____ am / pm Time on: ____:____ am / pm | |
| **Other comments:** |  | |

| **DAY 4:** | **Sedentary Time Questionnaire** | Date: _____/ _____ / _____ |
| --- | --- | --- |
| **Sitting for study** | How long were you sitting or lying down while studying today?   \|  \|  \| hours \|  \|  \| minutes \| \| --- \| --- \| --- \| --- \| --- \| --- \| | |
| **Sitting for work** | How long were you sitting or lying down at your workplace or working from home today?   \|  \|  \| hours \|  \|  \| minutes \| \| --- \| --- \| --- \| --- \| --- \| --- \| | |
| **Sitting for Transport** | How long were you sitting or lying down for transport today?   \|  \|  \| hours \|  \|  \| minutes \| \| --- \| --- \| --- \| --- \| --- \| --- \| | |
| **Television Viewing** | How long were you sitting or lying down to watch TV today?   \|  \|  \| hours \|  \|  \| minutes \| \| --- \| --- \| --- \| --- \| --- \| --- \| | |
| **Computer, Internet, Electronic Games** | How long were you sitting or lying down while using the computer today?   \|  \|  \| hours \|  \|  \| minutes \| \| --- \| --- \| --- \| --- \| --- \| --- \| | |
| **Sitting for leisurely reading** | How long were you sitting or lying down while reading during your leisure time today?   \|  \|  \| hours \|  \|  \| minutes \| \| --- \| --- \| --- \| --- \| --- \| --- \| | |
| **Sitting for eating** | How long were you sitting or lying down while eating and drinking today?   \|  \|  \| hours \|  \|  \| minutes \| \| --- \| --- \| --- \| --- \| --- \| --- \| | |
| **Sitting for socializing** | How long were you sitting or lying down to socialize with family and friends today?   \|  \|  \| hours \|  \|  \| minutes \| \| --- \| --- \| --- \| --- \| --- \| --- \| | |
| **Sitting for other purposes** | How long were you sitting or lying down today in other pursuits NOT including the time that you have already logged above?   \|  \|  \| hours \|  \|  \| minutes \| \| --- \| --- \| --- \| --- \| --- \| --- \| | |

Please take a moment to add up your total sitting time for today. Considering how many hours you were awake, does this amount of sitting make sense? If not, please make changes to reflect your true sitting time.

**Thank you for your time**

| **DAY 5:** | **ActivPAL log** | Date: _____/ _____ / _______ |
| --- | --- | --- |
| **Sleep** | What time did you wake up today? _____:____ am / pm  What time did you get out of bed? _____:____ am / pm | |
|  | What time will you get into bed? _____:____ am / pm  What time will you go to sleep today? _____:____ am / pm | |
| **University** | Did you carry out any university-related activity today (e.g., lectures, tutorials, meetings, independent study, completing assignments, reading, sending emails, etc)? Please record all the times you spent doing these activities: | **□ No □ Yes** |
|  | **Time Started Time Finished** | |
|  | : am / pm : am / pm  : am / pm : am / pm  : am / pm : am / pm  : am / pm : am / pm  : am / pm : am / pm  : am / pm : am / pm  : am / pm : am / pm | |
| **Monitor** | Did you remove your thigh monitor today for more than 15 minutes? | |
|  | **□ No □ Yes** *If yes, please note time off/on:*  Time off: ____:____ am / pm Time on: ____:____ am / pm  Time off: ____:____ am / pm Time on: ____:____ am / pm | |
| **Other comments:** |  | |

| **DAY 5:** | **Sedentary Time Questionnaire** | Date: _____/ _____ / _____ |
| --- | --- | --- |
| **Sitting for study** | How long were you sitting or lying down while studying today?   \|  \|  \| hours \|  \|  \| minutes \| \| --- \| --- \| --- \| --- \| --- \| --- \| | |
| **Sitting for work** | How long were you sitting or lying down at your workplace or working from home today?   \|  \|  \| hours \|  \|  \| minutes \| \| --- \| --- \| --- \| --- \| --- \| --- \| | |
| **Sitting for Transport** | How long were you sitting or lying down for transport today?   \|  \|  \| hours \|  \|  \| minutes \| \| --- \| --- \| --- \| --- \| --- \| --- \| | |
| **Television Viewing** | How long were you sitting or lying down to watch TV today?   \|  \|  \| hours \|  \|  \| minutes \| \| --- \| --- \| --- \| --- \| --- \| --- \| | |
| **Computer, Internet, Electronic Games** | How long were you sitting or lying down while using the computer today?   \|  \|  \| hours \|  \|  \| minutes \| \| --- \| --- \| --- \| --- \| --- \| --- \| | |
| **Sitting for leisurely reading** | How long were you sitting or lying down while reading during your leisure time today?   \|  \|  \| hours \|  \|  \| minutes \| \| --- \| --- \| --- \| --- \| --- \| --- \| | |
| **Sitting for eating** | How long were you sitting or lying down while eating and drinking today?   \|  \|  \| hours \|  \|  \| minutes \| \| --- \| --- \| --- \| --- \| --- \| --- \| | |
| **Sitting for socializing** | How long were you sitting or lying down to socialize with family and friends today?   \|  \|  \| hours \|  \|  \| minutes \| \| --- \| --- \| --- \| --- \| --- \| --- \| | |
| **Sitting for other purposes** | How long were you sitting or lying down today in other pursuits NOT including the time that you have already logged above?   \|  \|  \| hours \|  \|  \| minutes \| \| --- \| --- \| --- \| --- \| --- \| --- \| | |

Please take a moment to add up your total sitting time for today. Considering how many hours you were awake, does this amount of sitting make sense? If not, please make changes to reflect your true sitting time.

**Thank you for your time**

| **DAY 6:** | **ActivPAL log** | Date: _____/ _____ / _______ |
| --- | --- | --- |
| **Sleep** | What time did you wake up today? _____:____ am / pm  What time did you get out of bed? _____:____ am / pm | |
|  | What time will you get into bed? _____:____ am / pm  What time will you go to sleep today? _____:____ am / pm | |
| **University** | Did you carry out any university-related activity today (e.g., lectures, tutorials, meetings, independent study, completing assignments, reading, sending emails, etc)? Please record all the times you spent doing these activities: | **□ No □ Yes** |
|  | **Time Started Time Finished** | |
|  | : am / pm : am / pm  : am / pm : am / pm  : am / pm : am / pm  : am / pm : am / pm  : am / pm : am / pm  : am / pm : am / pm  : am / pm : am / pm | |
| **Monitor** | Did you remove your thigh monitor today for more than 15 minutes? | |
|  | **□ No □ Yes** *If yes, please note time off/on:*  Time off: ____:____ am / pm Time on: ____:____ am / pm  Time off: ____:____ am / pm Time on: ____:____ am / pm | |
| **Other comments:** |  | |

| **DAY 6:** | **Sedentary Time Questionnaire** | Date: _____/ _____ / ______ |
| --- | --- | --- |
| **Sitting for study** | How long were you sitting or lying down while studying today?   \|  \|  \| hours \|  \|  \| minutes \| \| --- \| --- \| --- \| --- \| --- \| --- \| | |
| **Sitting for work** | How long were you sitting or lying down at your workplace or working from home today?   \|  \|  \| hours \|  \|  \| minutes \| \| --- \| --- \| --- \| --- \| --- \| --- \| | |
| **Sitting for Transport** | How long were you sitting or lying down for transport today?   \|  \|  \| hours \|  \|  \| minutes \| \| --- \| --- \| --- \| --- \| --- \| --- \| | |
| **Television Viewing** | How long were you sitting or lying down to watch TV today?   \|  \|  \| hours \|  \|  \| minutes \| \| --- \| --- \| --- \| --- \| --- \| --- \| | |
| **Computer, Internet, Electronic Games** | How long were you sitting or lying down while using the computer today?   \|  \|  \| hours \|  \|  \| minutes \| \| --- \| --- \| --- \| --- \| --- \| --- \| | |
| **Sitting for leisurely reading** | How long were you sitting or lying down while reading during your leisure time today?   \|  \|  \| hours \|  \|  \| minutes \| \| --- \| --- \| --- \| --- \| --- \| --- \| | |
| **Sitting for eating** | How long were you sitting or lying down while eating and drinking today?   \|  \|  \| hours \|  \|  \| minutes \| \| --- \| --- \| --- \| --- \| --- \| --- \| | |
| **Sitting for socializing** | How long were you sitting or lying down to socialize with family and friends today?   \|  \|  \| hours \|  \|  \| minutes \| \| --- \| --- \| --- \| --- \| --- \| --- \| | |
| **Sitting for other purposes** | How long were you sitting or lying down today in other pursuits NOT including the time that you have already logged above?   \|  \|  \| hours \|  \|  \| minutes \| \| --- \| --- \| --- \| --- \| --- \| --- \| | |

Please take a moment to add up your total sitting time for today. Considering how many hours you were awake, does this amount of sitting make sense? If not, please make changes to reflect your true sitting time.

**Thank you for your time**
