# Supplementary material for: Feasibility of Reducing and Breaking Up University Students' Sedentary Behaviour: Pilot Trial and Process Evaluation
Source: Front Psychol. 2021 Jun 10;12:661994. doi: 10.3389/fpsyg.2021.661994 (PMC8222591; doi:10.3389/fpsyg.2021.661994)
Supplement: Supplementary file 4 [file Table_4.DOCX]

**Supplementary file 4**: Interview schedule for process evaluation

1. Do you feel you’ve changed your sitting patterns during the past week? How much have you reduced your daily sitting time (e.g., 30 mins, 1 hour)? And in terms of prolonged sitting (e.g., frequency of breaks)? How have you replaced sitting (e.g., walking, standing)?

(If you were not able to introduce changes in your sitting patterns, why do you think this happened? What would need to change for you to reduce and break up your sitting time?)

2. What strategies have you implemented to reduce and break up your sitting time (i.e., what changes have you made in your daily routines)? (How and what worked and what didn’t) What helped you to change your behaviour (facilitators)? What prevented you from reducing and breaking up sitting (barriers)?

3. We are interested in knowing why and how you’ve changed your sitting patterns. In order to explore potential mechanisms of change, I’m going to give you this document, which reflects different reasons for reducing and breaking up sitting expressed by university students.

Could you please indicate, on a scale of 0-10, how much do you think these sentences apply to your own change process during the past week? With 0 meaning that the specific reason for change doesn’t apply to you at all and 10 meaning that it completely reflects your reason for change.

4. The following questions refer to the intervention itself. We would like to know your opinion on the different intervention components, so we can improve the design and make additional changes if necessary. Would you change something about the intervention? What? Why?

Let’s go through each component. Could you think about (i) how well (or bad) was it delivered/presented (e.g., clarity), and (ii) how useful (or irrelevant) was it for your own behaviour change process?

- - Information provided in the face-to-face session and intervention booklet.
  - Booklet activities (feedback, pros/cons exercise, suggested strategies).
  - Daily text messages to reduce and break up sitting. Were they ‘invasive’ to some extent?
  - Materials provided (poster, apps). Did you use any? What was your experience using them?

5. To wrap up, we would like to know more about potential factors, external to the study, that have influenced your sitting patterns during the last two weeks (e.g., weather, high workload). Where the two weeks ‘comparable’?

6. Are there certain periods of the academic year where your sitting patterns are different? Would you say sitting is ‘seasonal’?

7. For what type of activity was it easiest to change your sitting patterns? For what type of activities was it the hardest? (Occupational vs non-occupational; reduce sitting vs break up sitting)

8. Has taking part in the study had any other effects, apart from sitting time?

9. What do you think it will happen to your sitting patterns in the future?
