# Supplementary material for: Feasibility of Reducing and Breaking Up University Students' Sedentary Behaviour: Pilot Trial and Process Evaluation
Source: Front Psychol. 2021 Jun 10;12:661994. doi: 10.3389/fpsyg.2021.661994 (PMC8222591; doi:10.3389/fpsyg.2021.661994)
Supplement: Supplementary file 5 [file Table_5.DOCX]

**Supplementary file 5:** Mechanisms of action questionnaire (statements).

| **Reason for change: I’ve changed my sitting patterns over the past week because…** |
| --- |
| I’ve learnt more about this particular behaviour and why time spent sitting matters  *It doesn’t apply to me* **0 1 2 3 4 5 6 7 8 9 10** *It applies to me* |
| I have now a more negative attitude towards *too much* sitting and a more positive one towards reducing and breaking up my sitting time  *It doesn’t apply to me* **0 1 2 3 4 5 6 7 8 9 10** *It applies to me* |
| I’m now aware of the negative consequences of sitting  *It doesn’t apply to me* **0 1 2 3 4 5 6 7 8 9 10** *It applies to me* |
| I’ve developed a conscious decision to reduce and break up my sitting time  *It doesn’t apply to me* **0 1 2 3 4 5 6 7 8 9 10** *It applies to me* |
| I’ve used reminders to reduce and break up my sitting time (e.g., posters, alarms, apps)  *It doesn’t apply to me* **0 1 2 3 4 5 6 7 8 9 10** *It applies to me* |
| I’ve used self-regulation strategies to reduce and break my sitting time (e.g., set specific goals, tracking my own behaviour)  *It doesn’t apply to me* **0 1 2 3 4 5 6 7 8 9 10** *It applies to me* |
| I’m now more motivated to reduce and break up my sitting time  *It doesn’t apply to me* **0 1 2 3 4 5 6 7 8 9 10** *It applies to me* |
| I’ve received feedback on how much time I spent in total and prolonged sitting  *It doesn’t apply to me* **0 1 2 3 4 5 6 7 8 9 10** *It applies to me* |
| I’ve developed new skills to reduce and break up my sitting time  *It doesn’t apply to me* **0 1 2 3 4 5 6 7 8 9 10** *It applies to me* |
| I consider now reducing and breaking up sitting as part of my role as university student  *It doesn’t apply to me* **0 1 2 3 4 5 6 7 8 9 10** *It applies to me* |
| I’ve used ‘prizes’ or incentives to reduce and break up my sitting time (e.g., snacks, during the breaks I’ve reminded myself of the benefits of interrupting sitting)  *It doesn’t apply to me* **0 1 2 3 4 5 6 7 8 9 10** *It applies to me* |
| I’ve realised that people within the university setting encourage and approve reducing and breaking up sitting time.  *It doesn’t apply to me* **0 1 2 3 4 5 6 7 8 9 10** *It applies to me* |
| Other people have helped me to reduce and break up my sitting time  *It doesn’t apply to me* **0 1 2 3 4 5 6 7 8 9 10** *It applies to me* |
| I now see myself as someone who ‘sits less and moves more’  *It doesn’t apply to me* **0 1 2 3 4 5 6 7 8 9 10** *It applies to me* |
